# Enhanced Photocatalytic Hydrogen Evolution Activity Driven by the Synergy Between Surface Vacancies and Cocatalysts: Surface Reaction Matters

**DOI:** 10.1002/advs.202407092

**Published:** 2024-09-25

**Authors:** Wenhui Yue, Ziwei Ye, Cong Liu, Zehong Xu, Lingzhi Wang, Xiaoming Cao, Hiromi Yamashita, Jinlong Zhang

**Affiliations:** ^1^ Key Laboratory for Advanced Materials Shanghai Engineering Research Center for Multi‐media Environmental Catalysis and Resource Utilization School of Chemistry and Molecular Engineering East China University of Science & Technology Shanghai 200237 China; ^2^ School of Chemistry and Chemical Engineering Shanghai Jiao Tong University Shanghai 200240 China; ^3^ Division of Materials and Manufacturing Science Graduate School of Engineering Osaka University Osaka 565‐0871 Japan

**Keywords:** hydrogen evolution reaction, photocatalysis, surface vacancies, water dissociation

## Abstract

The incorporation of defects and cocatalysts is known to be effective in improving photocatalytic activity, yet their coupled contribution to the photocatalytic hydrogen evolution process has not been well‐explored. In this study, We demonstrate that the incorporation of S vacancies and NiSe can contribute to the improvement of charge separation efficiency via the formation of a strong electric field within the bulk ZnIn_2_S_4_ (ZIS) and on its surface. More importantly, We also demonstrate that the synergy of S vacancies and NiSe benefits the overall hydrogen evolution activity by facilitating the H_2_O adsorption and dissociation process. This is particularly important for hydrogen evolution taking place under alkaline conditions where the proton concentration is low, allowing ZISv‐NiSe (containing abundant S vacancies) to outperform ZIS‐NiSe under alkaline conditions. In contrast, under acid conditions, since there are already sufficient amounts of protons available for reaction, the hydrogen evolution activity became governed by the hydrogen adsorption/desorption process rather than the H_2_O dissociation process. This leads to ZIS‐NiSe exhibiting higher activity than ZISv‐NiSe due to its more favorable hydrogen adsorption energy. The findings thus provide insights into how defect and cocatalyst modification strategies can be tailor‐made to improve hydrogen evolution activity under different pH conditions.

## Graphical abstract

In addition to the adsorption and desorption of hydrogen on photocatalyst surface, the dissociation of water is another kinetic step limiting the overall hydrogen evolution activity under alkaline conditions. In this study, a method is developed where the water dissociation step can be facilitated by coupling the vacancy and cocatalyst modification strategy to achieve enhanced hydrogen evolution activity under alkaline conditions.

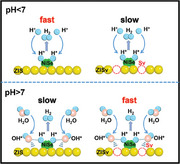

## 1 Introduction

Photocatalytic hydrogen evolution which allows direct conversion of solar light into clean and storable hydrogen gas is gaining increasing attention since it holds promise for addressing the ever‐worsening environmental and energy challenges.^[^ ^1^ ^]^ However, the performance of the current generation of photocatalysts is far from satisfactory for industrial applications due to both kinetic and/or thermodynamic limitations.^[^ ^2^ ^]^ To overcome these limitations, one strategy that has been explored extensively is the creation of vacancies on photocatalyst surface.^[^ ^3^ ^]^ Although vacancies are not always benign as they might serve as recombination centers, it is generally agreed that appropriate engineering of surface vacancies can be very efficient in improving photocatalytic activity, as they can alter the charge transfer process to improve charge separation efficiency, and provide active sites to improve surface reaction efficiency.^[^ ^4^ ^]^

In some particular cases, surface vacancies are also used as anchoring points for precise deposition of single atom or cluster cocatalysts.^[^ ^5^ ^]^ This is because these defective sites contain abundant unsaturated atoms, making it energetically more favorable to deposit cocatalysts precisely on these sites.^[^ ^6^ ^]^ Such selective deposition of cocatalysts on the vacancy sites can bring in many benefits, as vacancies can alter the surface electron structure which leads to unique electronic and catalytic properties for cocatalysts. For instance, Cao and co‐workers showed that by depositing single Au atoms selectively on Cd vacancies on the CdS photocatalyst, CO_2_ can be chemically bonded on the nearby Cd vacancies rather than single Au atoms to allow for more efficient activation of CO_2_.^[^ ^7^ ^]^ Zhang and co‐workers showed that the deposition of MoS_2_ quantum dots selectively on S vacancies of ZIS created intimate contact between them to allow more efficient migration of electrons from ZIS to MoS_2_, leading to high hydrogen evolution activity.^[^ ^8^ ^]^

It should be noted, however, that although surface vacancies can be filled in by cocatalysts which reduces their concentration, there are many cases where vacancies and cocatalysts coexist and each have their own role in contributing to the photocatalytic processes.^[^ ^9^ ^]^ In previous studies, the impact of vacancies and cocatalysts on the transportation and/or separation behavior of photogenerated charge carriers has been extensively explored. For instance, Su and co‐workers demonstrated that electrons that were generated upon excitation of ZIS could accumulate first on S vacancies and then transfer from S vacancies to Ti_3_C_2_T*_x_*, leading to improved electron transfer and separation efficiency.^[^ ^10^ ^]^ Similarly, Dai and co‐workers showed that the O vacancies on TiO_2_ surface can trap electrons generation upon TiO_2_ excitation and pass them on further to Pt via the formation of Pt─V_o_─Ti bonds to achieve more efficient transportation and separation of charge carriers.^[^ ^11^ ^]^ However, the impact of vacancies and cocatalysts on the surface reaction process has not been well explored. This has meant that the photocatalytic mechanism arrived at can be biased, which might lead to wrong principles for directing the design and synthesis of high‐performance photocatalysts.

In this study, we investigate the overall contribution of surface vacancies and cocatalysts to photocatalytic hydrogen evolution process, taking into consideration their influence on both charge transportation and/or separation behavior and on the surface reaction process. ZnIn_2_S_4_ photocatalysts containing abundant S vacancies (ZISv) and few S vacancies (ZIS) were synthesized using a hydrothermal method. NiSe as a non‐metal cocatalysts was then deposited on the surface of ZIS via a photo‐deposition method without significantly reducing the vacancy concentration. The results showed that incorporation of S vacancies and NiSe contributed to the improvement of charge transportation and separation efficiency via the formation of strong electric field in the bulk of ZIS photocatalysts and on their surface. More importantly, it was found that the coexistence of S vacancies and NiSe cocatalysts also facilitated the kinetic process for the adsorption of H_2_O molecules and their further dissociation on ZIS surface to generate more protons. As a result, under alkaline conditions where the proton concentration was low, ZISv‐NiSe exhibited higher hydrogen evolution activity compared to ZIS‐NiSe, despite having less favorable hydrogen adsorption energy. In contrast, under acid conditions where the proton concentration was high, the kinetic process for H_2_O dissociation was no longer the rate‐limiting step. Under such conditions, the hydrogen evolution activity became governed by the hydrogen adsorption‐desorption step on photocatalysts surface, which led to ZIS‐NiSe having higher activity than ZISv‐NiSe due to its more favourable hydrogen adsorption energy. This work thus highlights the critical role of vacancies and cocatalysts in promoting the surface reaction process and provides insights into how defect and cocatalyst engineering strategies can be effectively coupled to synthesize highly efficient hydrogen evolution photocatalysts.

## 2 Results and Discussion

**Figure 1:**
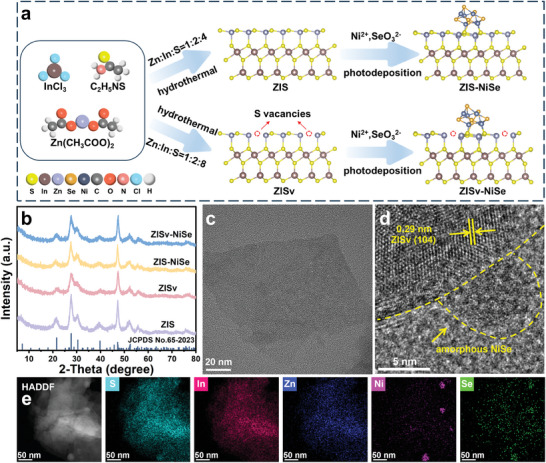
a) Schematic illustration of the synthetic process for the ZIS, ZISv, ZIS‐NiSe, and ZISv‐NiSe samples. b) XRD patterns of the ZIS, ZISv, ZIS‐NiSe, and ZISv‐NiSe samples. c) TEM image of the ZISv sample. d) HRTEM image of the ZISv‐NiSe sample. e) HAADF‐STEM and the corresponding elemental mapping images of the ZISv‐NiSe sample.

As shown in **Figure** **1**a, ZISv was synthesized using a previously reported hydrothermal method in the presence of excessive amount of Thioacetamide (TAA).^[^ ^12^ ^]^ Detailed explanations for the formation of S vacancies can be found in the supporting information. The same method was also used for synthesizing ZIS, except that the amount of TAA added was reduced by half during the synthesis. As shown in Figure 1b, the diffraction pattern for both samples agreed well with those of hexagonal ZIS. Specifically, the diffraction peak at 21.6°, 27.7°, 30.4°, 39.8°, 47.2°, 52.4°, 55.6°, and 76.4° corresponds to the (006), (102), (104), (108), (110), (116), (022) and (213) planes, respectively (JCPDS card No. 65–2023).^[^ ^13^ ^]^ The lattice fringe of the (006) and (104) planes was also observed in the high‐resolution transmission electron microscopy (HRTEM) images, indicating that ZISv was successfully synthesized (Figure S1, Supporting Information).

The morphology of the ZISv samples was then investigated using transmission electron microscopy (TEM) characterization. As shown in Figure 1c, the product ZISv took the form of nanoflakes but tended to stack together to form clusters. After the photodeposition step, energy‐dispersive X‐ray (EDX) mapping results showed that small NiSe nanoparticles can be found all over the ZISv surface (Figure 1e). However, no lattice fringe that corresponds to the crystalline NiSe was observed, indicating that these NiSe nanoparticles existed mainly in the amorphous form (Figure 1d; Figure S2, Supporting Information). This is in agreement with the Powder X‐ray diffraction (XRD) results, in which no diffraction peak that corresponds to NiSe was observed even at higher loading percent (Figure S3, Supporting Information). The same characterizations for the ZIS and ZIS‐NiSe samples showed that their morphology and crystalline structure were identical to those of the ZISv and ZISv‐NiSe samples (Figures S4 and S5, Supporting Information).

**Figure 2:**
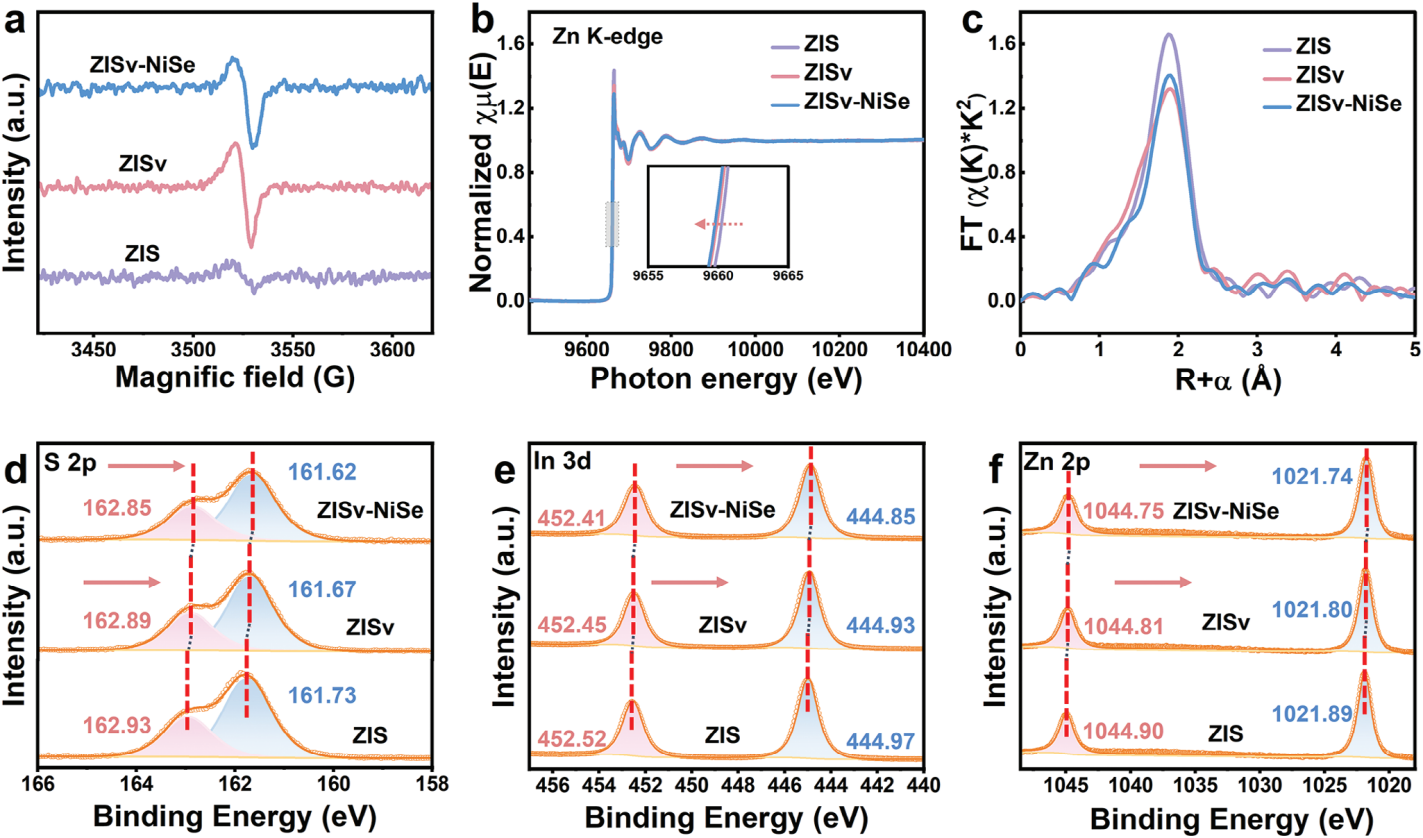
a) EPR spectra of the ZIS, ZISv, and ZISv‐NiSe samples. b) Normalized Zn K‐edge XANES spectra of the ZIS, ZISv, and ZISv‐NiSe samples. c) Fourier transforms *k* ^2^‐weighted of the EXAFS spectra for the ZIS, ZISv, and ZISv‐NiSe samples. d–f) High resolution XPS spectra of d) S 2p, e) In 3d, and f) Zn 2p for the ZIS, ZISv, and ZISv‐NiSe samples.

Inductively coupled plasma (ICP) analysis was then performed to determine the chemical composition of the as‐prepared samples. The results showed that the atomic ratio (Zn:In:S) for the ZISv sample was 1:2.1:3.2, which clearly deviated from the standard atomic ratio (1:2:4) for ZIS, indicating that the ZISv sample contained high concentration S vacancies. Consistent with the ICP results, the electron paramagnetic resonance (EPR) spectra showed that the ZISv sample gave a strong signal at g = 2.004, while the same signal was much weaker for the ZIS sample (**Figure** **2**a). Since the signal at g = 2.004 corresponds to the electrons captured by the S vacancies, the result also indicated that the ZISv sample contained more S vacancies compared to the ZIS sample.^[^ ^10^, ^14^ ^]^ To further confirm the existence of S vacancies, the local atomic structure of Zn was then investigated using X‐ray absorption near edge structure (XANES) analysis. As shown in Figure 2b,c, the K‐edge position of Zn for the ZISv sample shifted slightly towards the lower energy region compared to that observed for the ZIS sample. More importantly, detailed fitting of the extended X‐ray absorption fine structure (EXAFS) spectra showed that the intensity of the peak which corresponds to the first coordination shell was lower for the ZISv sample, confirming the existence of S vacancies.^[^ ^15^ ^]^ Of note is that although the intensity of the first coordination shell was slightly increased after the deposition of NiSe, it was still lower than that observed for the ZIS sample, suggesting that the S vacancy sites were largely retained after the deposition of NiSe. This is consistent with the EPR results which showed that the strong signal at g = 2.004 was also retained for the ZISv‐NiSe sample.

Further characterization of the as‐prepared samples was then performed with X‐ray photoelectron spectroscopy (XPS) analysis. For the ZIS sample, the characteristic peaks that correspond to the S 2p, In 3d, and Zn 2p, can be observed at 161.73 and 162.93 eV, 444.97 and 452.52 eV, 1021.89, and 1044.90 eV, respectively (Figure 2d–f). In comparison, since the existence of S vacancies increased electron density near the vacancy sites, these peaks shifted towards the lower binding energy region for the ZISv sample.^[^ ^16^ ^]^ For the ZISv‐NiSe sample, the presence of NiSe nanoparticles can be confirmed by the characteristic Se 3d and Ni 2p peaks observed at 54.16 and 54.98 eV, 856.28 and 873.67 eV, respectively (Figure S6b,c, Supporting Information). Additional peaks that can be assigned to Se^4+^ species were also observed at ≈58.87 eV. These peaks can be attributed to the commonly observed oxidized Se species since metal selenides are prone to oxidation in air atmosphere.^[^ ^17^ ^]^ Alternatively, they might also arise from the NiSeO_3_∙xSeO_3_^2‐^ precursor which did not fully convert to NiSe nanoparticles.^[^ ^18^ ^]^ However, control experiments showed that these Se^4+^ species only had a minor contribution to photocatalytic hydrogen evolution activity (Figure S7, Supporting Information). Detailed inspection of the XPS spectrum of the ZISv‐NiSe sample showed that the Zn 2p, In 3d, and S 2p peaks shifted further towards the lower binding energy region compared to those for the ZISv sample. This is due to the Fermi energy level of NiSe being higher than that of the ZISv, which leads to electron being transferred spontaneously from NiSe to ZISv until the equilibrium state is reached. An interfacial electric field was therefore formed at the interface pointing from NiSe to ZISv, which is expected to significantly promote charge separation efficiency since the interfacial electric field can provide a strong electrostatic driving force for photogenerated electron to be transferred from ZIS to NiSe, as we will discuss in the following sections.^[^ ^19^ ^]^

**Figure 3:**
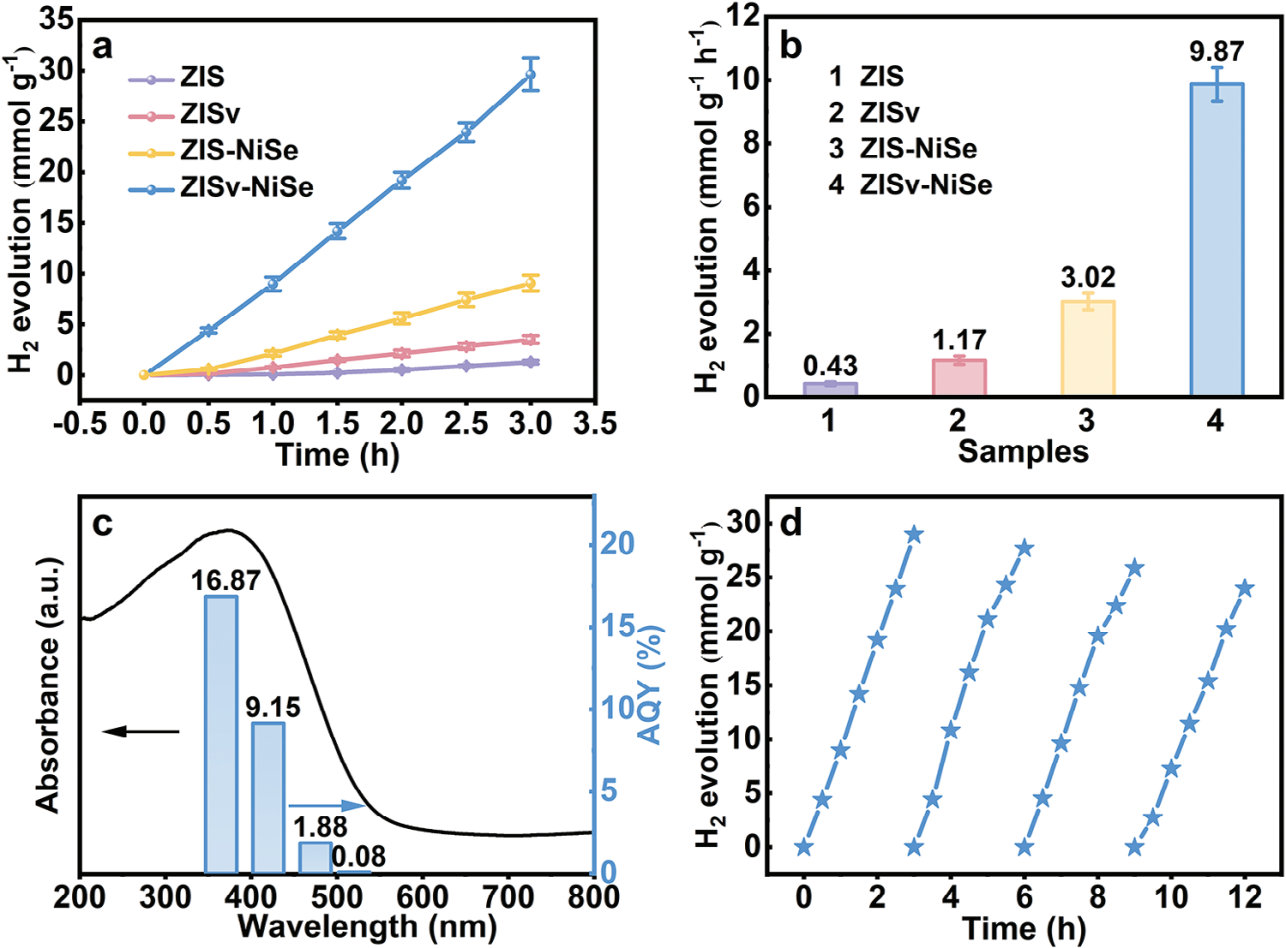
a) Plots illustrating the H_2_ evolution reaction at different time points for different samples. b) Histogram comparing the calculated H_2_ evolution rates for different samples. c) Calculated AQY plotted against wavelength for the ZISv‐NiSe sample. d) Plots showing H_2_ evolution activity of the ZISv‐NiSe sample at different test cycles.

Photocatalytic activity of the as‐prepared samples was tested in the aqueous solution of triethanolamine (TEOA) at pH = 10.9. As shown in **Figure** **3**a,b, the ZIS sample was almost inactive for hydrogen evolution, giving a low hydrogen evolution activity of ca. 0.43 mmol g^−1^ h^−1^. A ca. three‐fold increase in activity was observed for the ZISv sample, reaching ca. 1.17 mmol g^−1^ h^−1^. Much higher activity was observed for the ZISv‐NiSe and ZIS‐NiSe samples with NiSe nanoparticles deposited on their surface. At the optimal NiSe loading of 0.75 percent (Figure S8, Supporting Information), the ZISv‐NiSe sample exhibited an excellent hydrogen evolution rate of ca. 9.87 mmol g^−1^ h^−1^, which was more than 22 times higher than that measured for the ZIS sample. It is worth noting that the physical mixture of ZISv and NiSe showed considerably lower hydrogen evolution activity compared to the ZISv‐NiSe sample, underscoring the importance of forming close contact between ZISv and NiSe for achieving high activity (Figure S9, Supporting Information).

As shown in Figure 3c, the onset irradiation wavelength for hydrogen generation agreed well with the absorption edge for the ZISv‐NiSe sample, indicating that photocatalytic hydrogen evolution took place via bandgap transitions. The apparent quantum yield (AQY) measured at its maximum absorption of ca. 365 nm was determined to be 16.87%, which is higher than most of the state‐of‐the‐art photocatalysts (Tables S1 and S2, Supporting Information). Importantly, the ZISv‐NiSe sample also exhibited durability in repeated photocatalytic reaction cycles (Figure S10, Supporting Information). As shown in Figure 3d, its activity remained almost unchanged after an extended period of 12 h. The slight drop in activity might be due to the reconstruction of the ZISv surface which reduced the concentration of S vacancies (Figure S11, Supporting Information).

**Figure 4:**
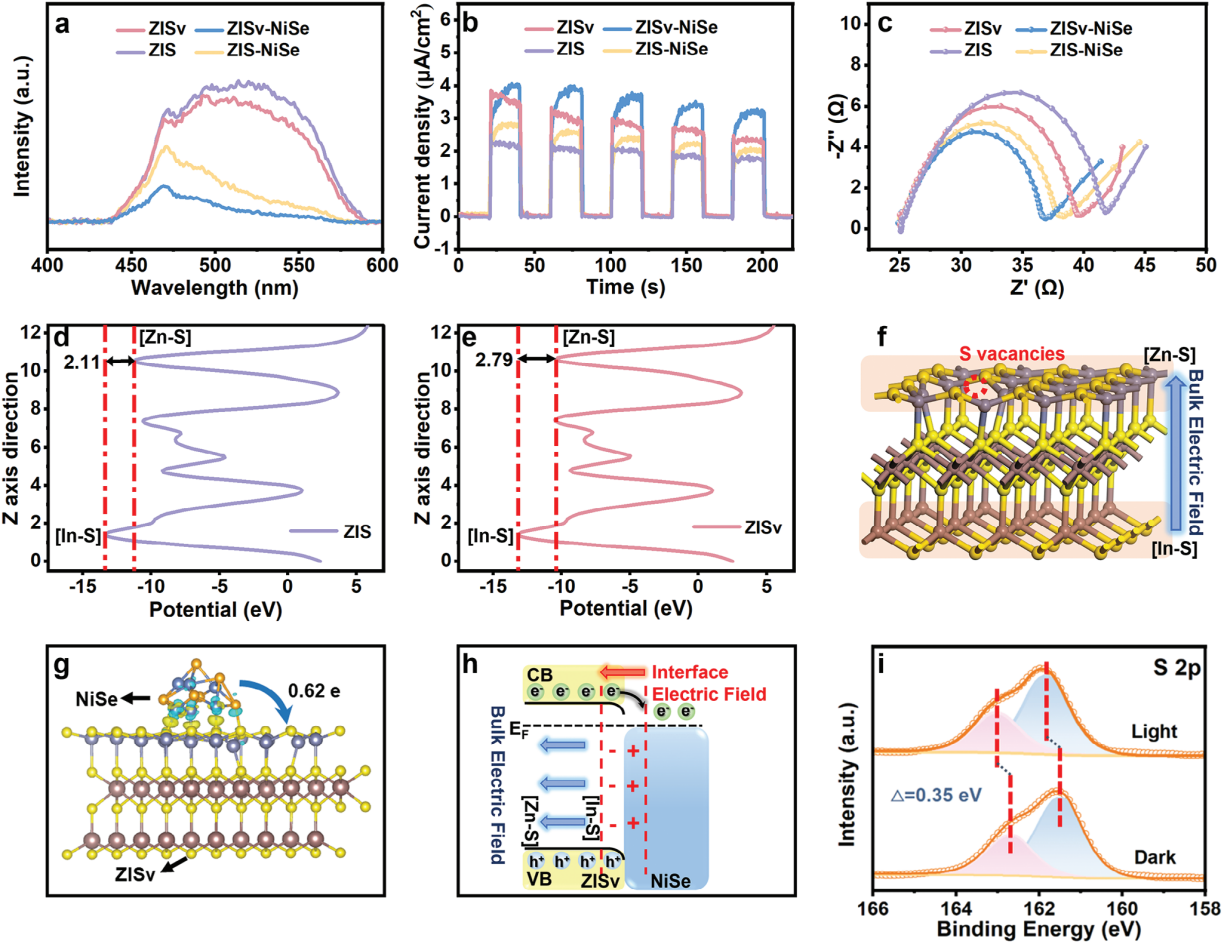
a–c) Photoluminescence emission spectra, transient photocurrent response, and electrochemical impedance spectra of the ZIS, ZISv, ZIS‐NiSe, and ZISv‐NiSe samples. d–e) DFT calculated the diagram of electrostatic potential along the [001] direction for ZIS and ZISv. f) DFT calculated diagram of crystal structure along the [001] direction for ZISv. g) Simulated differential charge density distribution at the interface between ZISv and NiSe (the yellow and blue areas represent electron accumulation and depletion, respectively). h) Schematic illustration of the bulk electric field strengthened by incorporation of S vacancies,  the interface electric field formed between ZISv and NiSe upon contact, and the charge transfer route between ZISv and NiSe upon irradiation. i) In situ XPS spectra of S 2p for the ZISv‐NiSe sample in the dark and under UV‐light irradiation.

The photochemical properties of as‐prepared samples were then studied to understand the contribution of S vacancies and NiSe nanoparticles in the current photocatalytic system. As shown in **Figure** **4**a, the photoluminescence (PL) spectrum of the ZIS sample gave a broad and intense emission peak under excitation of 350 nm light, indicating that electron–hole pairs underwent severe recombination upon generation. In comparison, a weaker emission peak was observed for the ZISv sample, since S vacancies can act as electron traps that capture photoelectrons upon their generation to improve separation efficiency.^[^ ^12^ ^]^ More importantly, density functional theory (DFT) calculation revealed that the introduction of S vacancies can also increase the charge distribution imbalance within the ZISv photocatalyst. As shown in Figure 4d–f, although an electrostatic potential difference of 2.11 eV already existed between the bulk and the surface layer for the ZIS sample, the potential difference was increased to 2.79 eV after the introduction of S vacancies, giving rise to a strengthened polarized electric field within the ZISv sample. This strong electric field can resist the Coulomb attraction between electrons and holes to improve their separation efficiency, accounting for the weaker emission signals observed for the ZISv sample in the PL spectrum.^[^ ^20^ ^]^ We then performed Kelvin‐probe force microscopy characterization under the dark and light conditions to gain deeper insights into the influence of this bulk electric field on the behaviors of photogenerated charge carriers. As shown in Figure S12 (Supporting Information), the surface potential difference under the dark and light conditions for the ZISv sample was much higher compared to that for the ZIS sample, indicating the existence of a stronger electric field for driving more efficient separation of photogenerated charge carriers for the ZISv sample.^[^ ^21^ ^]^ As a result of this strong bulk electric field, the ZISv sample also exhibited better performance in the photoelectrochemical characterizations, where the ZISv sample also gave stronger photocurrent response and lower resistance for transportation of charge carriers (Figure 4b,c).

In comparison with the ZISv samples, much weaker PL signal was observed for the ZISv‐NiSe samples under identical excitation conditions. The suppressed PL signal is an indication that photogenerated electrons transferred immediately from ZISv to NiSe, allowing electron‐hole pairs to be further separated. Consistent with the PL results, time‐resolved photoluminescence (TRPL) characterizations showed that the lifetime of the charge carriers was also prolonged after the introduction of NiSe (Figure S13 and Table S3, Supporting Information). As has been mentioned above, this is due to the formation of the interfacial electric field between ZISv and NiSe upon their contact. To confirm this, we measured the UV–vis diffuse reflection and valence‐band XPS spectra to determine the band structure of the ZIS and ZISv samples (Figure S14, Supporting Information). In addition, we also performed DFT calculation to investigate the charge distribution at the ZISv‐NiSe interface (Figures S15,S16, Supporting Information). As shown in Figure S16 (Supporting Information), the work function of ZISv was reduced from 6.17 to 5.06 eV after the introduction of NiSe, indicating that electrons transferred from NiSe to ZISv upon their contact.^[^ ^22^ ^]^ More direct evidence of this is that the NiSe side of the interface became electron deficient (highlighted in blue color) while the ZISv side of the interface became electron abundant (highlighted in yellow color). The total Bader number which represents the degree of charge transfer between the two was determined to be 0.62 e. Therefore, the overall result is that an interfacial electric field was formed between ZISv and NiSe upon forming contact (Figure 4h), giving rise to a significantly stronger photocurrent response and lower electrochemical impedance for the ZISv‐NiSe sample compared to the ZISv samples.

Due to the formation of electric field pointing from NiSe to ZISv, transfer of photoelectrons from ZISv to NiSe upon generation was facilitated. This is confirmed using in situ XPS analysis which showed that, the S 2p, In 3d, and Zn 2p peaks for the ZISv‐NiSe sample shifted towards the higher binding energy region (in comparison with that observed in the dark), indicating that electrons were indeed transferred from ZISv to NiSe upon excitation (Figure 4; Figure S17, Supporting Information).

**Figure 5:**
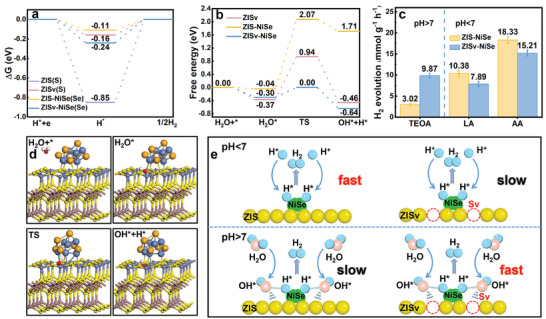
a) Calculated Gibbs free energy of H^*^ adsorption for the ZIS, ZISv, ZIS‐NiSe, and ZISv‐NiSe samples. b) Energy profiles for H_2_O dissociation on the ZISv, ZIS‐NiSe, and ZISv‐NiSe samples. c) Histogram comparing the calculated H_2_ evolution rates for ZIS‐NiSe and ZISv‐NiSe at different pH conditions using different sacrificial donors. d) Optimized structures for H_2_O dissociation taking place on the ZISv (001) surface in the presence of NiSe. e) Schematic diagram of hydrogen evolution reaction taking place on ZIS‐NiSe and ZISv‐NiSe surface under acidic and alkaline conditions.

However, although deposition of NiSe could lead to enhanced photoelectrochemical properties, it cannot account for the significantly larger activity difference observed between the ZISv‐NiSe sample and the ZIS sample when it comes to hydrogen evolution reaction. More specifically, the photocurrent response of the ZISv‐NiSe sample was only two times stronger than that observed for the ZIS sample, but the hydrogen evolution activity of the ZISv‐NiSe sample was more than 20‐fold that of the ZIS sample. Adding to this, the photocurrent response of the ZIS‐NiSe sample was even slightly lower than that of the ZISv sample, while the hydrogen evolution activity of the ZIS‐NiSe sample was much higher. This implies that NiSe must also contribute to the enhancement of hydrogen evolution activity by promoting the surface reaction process, and that this might be the main contribution of NiSe in photocatalytic hydrogen evolution reaction in the current system. To investigate this contribution, we start off by simulating the hydrogen adsorption and desorption process on the catalyst surface using DFT calculation. The ZIS (001) surface was taken as the representative catalyst surface during the DFT calculation. As shown in **Figures** **5**a and S18 (Supporting Information), hydrogen adsorbs strongly at the S site of the clean ZIS (001) surface with a hydrogen (H*) adsorption energy value of −0.85 eV, which means that it is difficult for hydrogen to desorb from ZIS surface to proceed with the evolution reaction. As a result, the pristine ZIS with low S vacancies concentration exhibited low hydrogen evolution activity. In comparison, the Se atoms for the ZIS‐NiSe sample make far better sites for hydrogen evolution reaction with a significantly weakened hydrogen adsorption energy of −0.11 eV. This, along with the improved charge separation efficiency for the ZIS‐NiSe sample, was the reason why it gave significantly higher hydrogen evolution activity than the ZIS sample.

In the presence of S vacancies, however, the hydrogen adsorption energy at the adjacent S site on the ZIS (001) surface was lowered considerably from −0.85 to −0.16 eV. This makes the adjacent S site a potential active site for hydrogen evolution reaction and enables the ZISv sample to exhibit higher hydrogen evolution activity than the ZIS sample. In contrast, the hydrogen adsorption energy at the Se site on NiSe nanoparticles was increased from −0.11 to −0.24 eV with S vacancies on ZIS surface. On the basis of Sabatier's principle, one would expect that the ZISv‐NiSe sample should exhibit lower hydrogen evolution activity than the ZIS‐NiSe sample.^[^ ^23^ ^]^ Clearly, this does not agree with the hydrogen evolution activity observed for these two samples and indicates that adsorption and desorption of hydrogen might not be the only kinetic step determining the activity of hydrogen evolution under the current reaction conditions.

To account for this observation, we take into consideration the water dissociation step which is known to be another rate‐limiting step for hydrogen evolution taking place under alkaline conditions.^[^ ^24^ ^]^ As shown in Figure S19 (Supporting Information), the free adsorption energy of water ($\Delta G_{H_{2}O*}$) on the clean ZIS (001) surface was calculated to be 0.00 eV, suggesting that H_2_O molecules cannot adsorb spontaneously on the ZIS (001) surface. Moreover, dissociation of H_2_O on the clean ZIS (001) surface was thermodynamically unfavorable since it was a strong endothermic process which requires a high energy input of 1.21 eV (Δ*E* = 1.21 eV) (Figure S20, Supporting Information), which is another reason why the ZIS sample exhibited lowest hydrogen evolution amongst all. In comparison, with S vacancies on the ZIS (001) surface, the Zn sites became coordinative unsaturated, making it energetically favorable for H_2_O to adsorb (Figure S21, Supporting Information) via the formation of the Zn─O bond. The strong interaction between Zn and O can also be confirmed using the crystal orbital Hamilton population (COHP) calculation. As shown in Figure S22 (Supporting Information), the ‐ICOHP value for the ZISv sample was greater than that for the ZIS sample, indicating that OH can interact strongly with ZISv surface via the formation of Zn─O bond.^[^ ^25^ ^]^ However, dissociation of H_2_O molecules was still kinetically prohibited since OH cannot be stably adsorbed on the S vacancy at the transition state to capture the hydrogen by the S site (Figure S14a,S23a, Supporting Information). Therefore, a formidable energy barrier of 1.31 eV is still required for H_2_O dissociation for the ZISv sample (Figure 5b), accounting for its second lowest hydrogen evolution activity among all.

In the case when S vacancies and NiSe nanoparticles coexist on ZIS surface, NiSe nanoparticles can interact with the H species in the transition state, while S vacancies can provide stable adsorption sites for OH species (Figure S23c, Supporting Information). This stabilization lowers the overall energy required for dissociation. Therefore, the energy barrier for H_2_O dissociation was reduced by more than 1 eV, allowing H_2_O molecules to dissociate easily to produce hydrogen intermediates (Figure 5b,d). These hydrogen intermediates then adsorb on the nearby Se sites where they recombined to generate molecular hydrogen, permitting high hydrogen evolution activity for the ZISv‐NiSe sample. Without S vacancies, however, the H_2_O dissociation process on the ZIS (001) surface remained a thermodynamically unfavorable process even in the presence of NiSe nanoparticles (Figure 5b; Figure S24, Supporting Information). More importantly, it was also a kinetically unfavorable process with an energy barrier of 2.11 eV, making it difficult for water to dissociate on the ZIS‐NiSe sample. This explains why the ZIS‐NiSe sample exhibited lower hydrogen evolution activity than the ZISv‐NiSe sample, despite having a more favorable hydrogen adsorption energy on its surface.

To verify this proposed mechanism, we performed control experiments in an aqueous solution of lactic acid (pH = 1.6) and ascorbic acid (pH = 2.4) for hydrogen evolution reaction. Under such conditions, dissociation of H_2_O became less important since there were already sufficient amounts of protons available to proceed the hydrogen evolution reaction. Therefore, it is expected that adsorption and desorption of hydrogen was the main kinetic process dictating the activity of the photocatalysts towards hydrogen evolution. Indeed, as shown in Figure 5c, although the absolute hydrogen evolution activity of the ZISv‐NiSe and ZIS‐NiSe samples had changed compared to that under alkaline conditions, the ZIS‐NiSe sample exhibited higher hydrogen evolution activity due to its more favorable hydrogen adsorption energy. For comparison, we also investigated the performance of the ZIS and ZISv samples under acid conditions. As expected, the ZISv sample outperformed the ZIS sample due to its enhanced charge separation efficiency and more favorable hydrogen adsorption energy (Figure S25, Supporting Information). Moreover, it is also interesting to have found out that the hydrogen evolution activity of the ZIS‐NiS and ZISv‐NiS samples followed the same pattern under different pH conditions, possibly due to the similar physiochemical properties of NiSe and NiS (Figure S26, Supporting Information).^[^ ^26^ ^]^

The overall mechanism accounting for the activity difference for ZISv‐NiSe and ZIS‐NiSe samples under different pH conditions is summarized in Figure 5e. Under acidic conditions where there is sufficient amount of proton available for evolution reaction, adsorption and desorption of hydrogen was the kinetic step determining the overall hydrogen evolution activity. Due to its more favorable hydrogen adsorption energy, the ZIS‐NiSe sample exhibited higher hydrogen evolution activity compared to the ZISv‐NiSe sample. However, under alkaline conditions, the kinetic step limiting the overall hydrogen evolution activity became the H_2_O dissociation step since there is not a sufficient amount of proton available for reaction. Under such conditions, the ZISv‐NiSe sample exhibited higher hydrogen evolution activity compared to the ZIS‐NiSe sample since it allowed for more efficient dissociation of H_2_O on its surface.

## 3 Conclusion

To sum up, we demonstrated in this work the critical role of the kinetic steps for the surface reaction process in governing the overall photocatalytic hydrogen evolution activity. On the basis of this understanding, we proposed a method where we coupled effectively the vacancy and cocatalyst modification strategies to achieve high hydrogen evolution activity. Specifically, the incorporation of NiSe cocatalysts and S vacancies on ZIS surface enabled the formation of electric field which improved charge transportation/separation efficiency. More importantly, the existence of NiSe cocatalysts and nearby S vacancies also promoted the H_2_O dissociation step for generating hydrogen intermediates for proceeding hydrogen evolution reaction under alkaline conditions. The concept demonstrated here provides insights for coupling the vacancy and cocatalyst modification strategies for synthesizing highly efficient hydrogen evolution photocatalyst under different pH conditions, and calls for attention to the surface reaction process which is often overlooked for photocatalytic hydrogen evolution.

## 4 Experimental Section

### Synthesis of ZIS

Zn(CH_3_COO)_2_·2H_2_O (0.4 mmol), InCl_3_·4H_2_O (0.8 mmol), and TAA (1.6 mmol) were dissolved in 15 mL deionized water and 15 mL absolute ethanol with vigorous stirring for 30 min. Then, the mixture was transferred into a 100 mL Teflon‐lined autoclave and heated at 180 °C for 24 h. After cooling down to room temperature, the obtained products were collected by centrifugation, washed several times with deionized water and ethanol, and dried at 70 °C in air.

### Synthesis of ZISv

The procedure of ZISv is similar to that of ZIS except that the raw material TAA was added to the solution at 3.2 mmol.

### Synthesis of ZISv‐NiSe

ZISv (40 mg) was added to 40 mL anhydrous ethanol solution, followed by 52 µL Ni(NO_3_)_2_·6H_2_O (0.1 m). Five minutes later, 104 µL Na_2_SeO_3_ (0.075 m) solution was added. After bubbling with N_2_ for 10 min, the above solution was illuminated by a 300 W Xenon lamp with a 420 nm cut‐off filter for 10 min to induce the deposition of NiSe on ZISv surface. Finally, the products were filtrated, rinsed, and dried at 70 °C to obtain the ZISv‐NiSe photocatalysts. (ZISv‐xNiSe, x% represents the mass ratio of Ni to ZISv). ZIS‐NiSe was also prepared by the same procedure, except that ZIS was added. The physical mixture of ZISv and NiSe was prepared by grinding 200 mg of ZISv and 3.6 mg of NiSe together.

### Photocatalytic Hydrogen Evolution

Photocatalytic H_2_ evolution reaction was conducted using an online Labsolar‐6A system (Beijing Perfectlight Technology Co., Ltd., China). The catalyst powder (10 mg) was dispersed in 100 mL of a 10 vol.% TEOA‐water solution (100 mL of a 10 vol.% DL‐Lactic acid‐water solution or 100 mL of aqueous solution containing 0.2 m ascorbic acid) in a top‐irradiation quartz vessel. The reactor was maintained at 5 °C using cooling water. Prior to light irradiation, the system was vacuumed for 10 min to remove any residual air. The amount of gas was detected in situ through an online gas chromatograph (SP‐7890plus, Shandong lunanruihong chemical engineering instruments Co., Ltd., China) with Ar as carried gas to determine the amount of produced H_2_. The H_2_ evolution rates were the average of three separate measurements.

### Characterizations

The characterization methods and DFT calculation are supplemented in the Supporting Information.

## Conflict of Interest

The authors declare no conflict of interest.

## Supporting information

Supporting Information

## Data Availability

The data that support the findings of this study are available in the supplementary material of this article.
