# Supplementary material for: Enhanced Photocatalytic Hydrogen Evolution Activity Driven by the Synergy Between Surface Vacancies and Cocatalysts: Surface Reaction Matters
Source: Adv Sci (Weinh). 2024 Sep 25;11(43):2407092. doi: 10.1002/advs.202407092 (PMC11578326; doi:10.1002/advs.202407092)
Supplement: Supplementary file 1 — Supporting Information [file ADVS-11-2407092-s001.docx]

Supporting Information

Enhanced photocatalytic hydrogen evolution activity driven by the synergy between surface vacancies and cocatalysts: Surface reaction matters

Wenhui Yue^a#^, Ziwei Ye^a#^*, Cong Liu^#^, Zehong Xu^a^, Lingzhi Wang^a^, Xiaoming Cao^b^**, Hiromi Yamashita^c^, Jinlong Zhang^a^***

Experimental Section

**Chemicals:** Zn(CH_3_COO)_2_·2H_2_O (99.8%), absolute ethanol (99.7%), InCl_3_·4H_2_O (99%), Nickel selenide (NiSe, 99.5%), triethanolamine (TEOA, 98%), DL-Lactic acid (85-90%), and thioacetamide (TAA, 99.8%) were obtained from Shanghai Macklin Biochemical Technology Co., Ltd. Ascorbic acid (99%) and Na_2_SO_4_ (99%) were purchased from Shanghai Aladdin Biochemical Technology Co., Ltd. Ni(NO_3_)_3_·6H_2_O (98%) and Na_2_SeO_3_ (98%) were purchased from Sinopharm Chemical Reagent Co., Ltd. 5,5-Dimethyl-1-pyrroline N-oxide (DMPO, 98%) was purchased from Shanghai Titan Scientific Co.,Ltd. All the materials were used without further purification. Double-deionized water with a conductivity of 18.25 MΩ·cm was used throughout the experiments.

**Characterizations:** Powder X-ray diffraction (XRD) characterization was performed on a Rigaku D/MAX 2550 with Cu K radiation (λ = 1.5406 Å), operated at 40 kV and 40 mA in the 5-80° (2θ) range. Transmission electron microscope (TEM) and energy-dispersive spectroscopy (EDS) characterizations were performed on a Talos F200X transmission electron microscope. The binding energies of Zn, In, S, Ni, and Se were performed on the K-Alpha X-ray photoelectron spectrometer system (XPS, Thermo Fisher Scientific). All of the binding energies were calibrated to the C 1 s peak of adventitious surface carbon at 284.8 eV. The electron paramagnetic resonance spectra (EPR) were recorded on a Bruker 100G-18KG/EMX-8/2.7 X-band spectrometer.

A Bruker Dimension Icon atomic force microscope (AFM) was employed to measure the surface potentials of different samples under Kelvin probe force microscopy (KPFM) mode. The amount of Zn, In and S in ZIS, ZISv and ZISv-0.75NiSe were measured by inductively coupled plasma mass spectrometry (ICP-MS). UV-Visible diffuse reflectance spectroscopy (UV-vis DRS) measurement was performed on a UV 3600 instrument (Shimadzu). Photoluminescence (PL) emission spectra were acquired on a fluorescence spectrometer (Hitachi F-4600) with a 350 nm excitation wavelength. Time-resolved photoluminescence (TRPL) decay spectra were acquired on an FLS1000 fluorescence lifetime spectrophotometer with a 367 nm excitation wavelength and a 460 nm emission wavelength. In situ irradiated X-ray photoelectron spectroscopy (ISI-XPS) was carried out on the X-ray photoelectron spectroscopy (ESCALAB 250Xi, Thermo Scientific). During the test, binding energies of the as-prepared photocatalysts without light irradiation was characterized with all lights off. And then, binding energies of the as-prepared sample was tested under the irradiation of a 300 W Xe lamp with AM1.5 cutoff filter. Photoelectrochemical test was conducted using an electrochemical workstation (Zahner, Zennium) with a three-electrode system. The working electrode was a fluorine-doped tin oxide (FTO) glass plate coated with photocatalysts, which was prepared by dropping 0.02 mL of photocatalyst dispersion (10 mg of photocatalyst dispersed in 1 mL of ethanol) on the FTO glass plate. A Pt wire and a saturated calomel electrode were used as the counter electrode and reference electrode, respectively. The electrolyte solution for the electrochemical impedance spectroscopy (EIS) measurement contained 25 mM K_3_Fe(CN)_6_, 25 mM K_4_Fe(CN)_6_, and 0.1 M KCl. During the EIS measurement, the amplitude of the measurement was set to be 10 mV and the frequency range was set between 10^-1^ and 10^5^ Hz. The photocurrent density measurement and Mott-Schottky plot were performed in a 0.5 M Na_2_SO_4_ solution. XAFS measurements conditions. Zn K-edge XAFS analyses were performed with Si (311) crystal monochromators at the BL14W Beam line at the Shanghai Synchrotron Radiation Facility (SSRF) (Shanghai, China). The analysis of the XAFS spectra were conducted using the Demeter software package (ATHENA and ARTEMIS, respectively).^[1]^ All fits were performed in the R space with a k-weight of 2.

**Photocatalytic measurement:** The wavelength for measuring the apparent quantum efficiency (AQY) was set to be 365, 420, 475, and 520 nm. AQY can be calculated using the following function.

$$\begin{aligned} \mathrm{AQY}\left( \% \right)=\frac{Number of reacted electrons}{Total number of incident photons}\times100\#\left( 1 \right) \end{aligned}$$

$$\begin{aligned} =\frac{2\times The Number of evoluted H_{2} electrons}{Total number of incident photons}\times100\#\left( 2 \right) \end{aligned}$$

**DFT computational details:** All the spin-polarized density functional theory calculations were carried out using the Vienna ab initio Simulation Package (VASP).^[2]^ Electronic exchange and correlation were treated within the generalized gradient approximation (GGA) by using the Perdew-Burke-Ernzerhof (PBE) function.^[3]^ PBE-D3 (BJ) is also included to describe van der Waals interaction.^[4]^ The plane wave energy cutoff was 450 eV. The convergence criteria for the electronic structure and the geometry were 10^-5^ eV and 0.05 eV/Å, respectively. The dipole correction was performed throughout the calculations to take the polarization effect into account. The exposed (001) surface of ZIS observed in the experiments were modeled by a 4-layer *p*(3$\times$3) unit cell of 15.6 Å, 15.5 Å. The vacuum space was set at 15 Å in the z direction. During the geometry optimization, the bottom one layers were fixed while the atomic positions of the adsorbates and the top three layers were relaxed. The 2$\times$2$\times$1 Monkhorst–Pack k-point mesh was used for ZIS (001). The ZISv-NiSe was modeled with the NiSe cluster consisting of 6 Ni atoms and 6 Se atoms anchored on the ZISv (001) surface.

**The Gibbs free energy was calculated as follows:**

ΔG= ΔE + ΔZPE − ΔTS (where E is the calculated total energy of the surface slab with adsorbed atoms, ZPE is the zero-point vibrational enthalpy, and TS is the entropy.)

The formation mechanism for S vacancies in ZISv: As TAA decompose under the hydrothermal conditions, it releases H_2_S which can undergo further ionization to form HS⁻ and S²⁻ ions. While S²⁻ ions combine with Zn²⁺ and In³⁺ ions for the formation of ZIS, HS^-^ is a strong reducing agent which is capable of reducing some of the Zn²⁺ and In³⁺ ions. This leads to the generation of S vacancies on ZIS as the valence state of Zn and In ions are reduced.


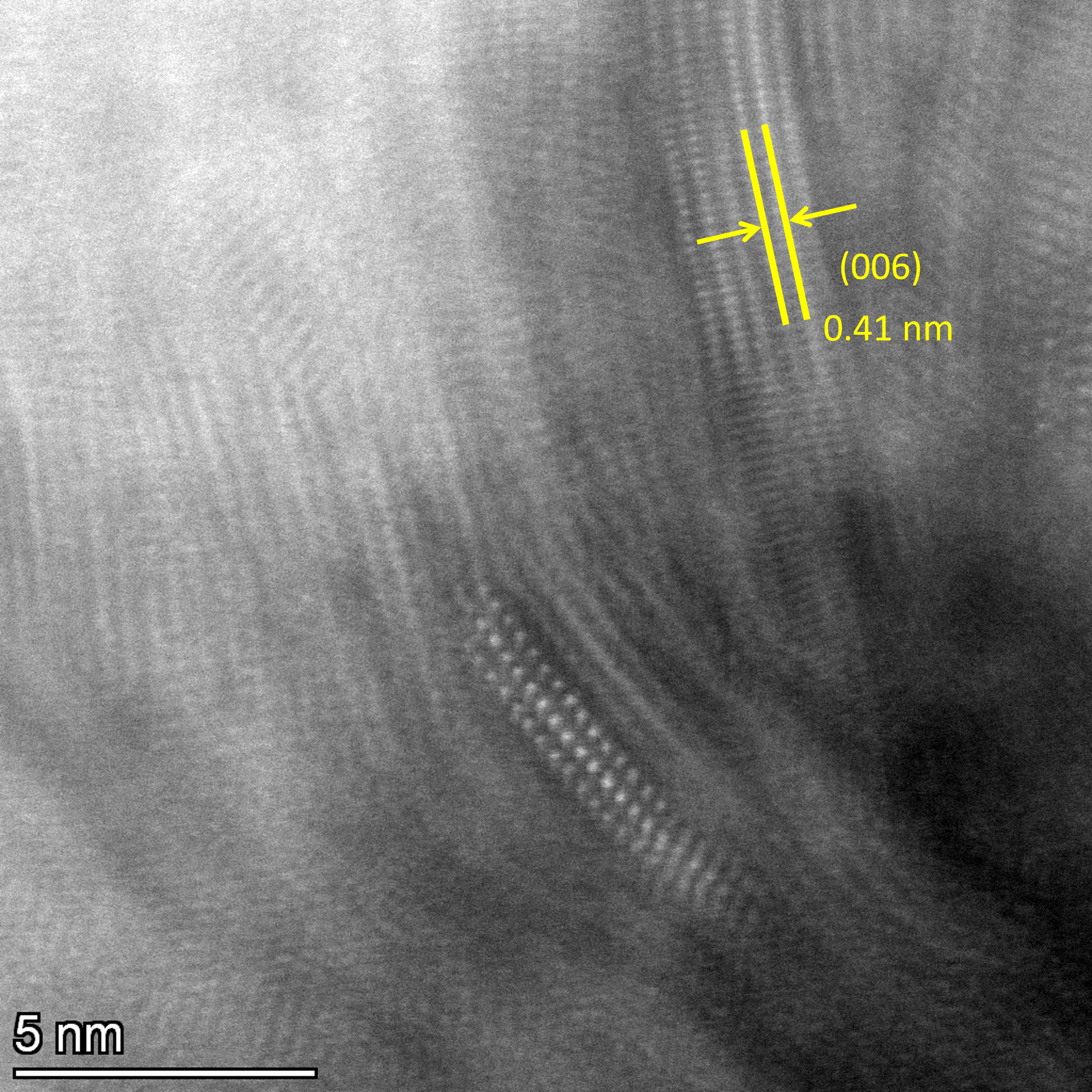


**Figure S1.** HRTEM image of the ZISv sample.


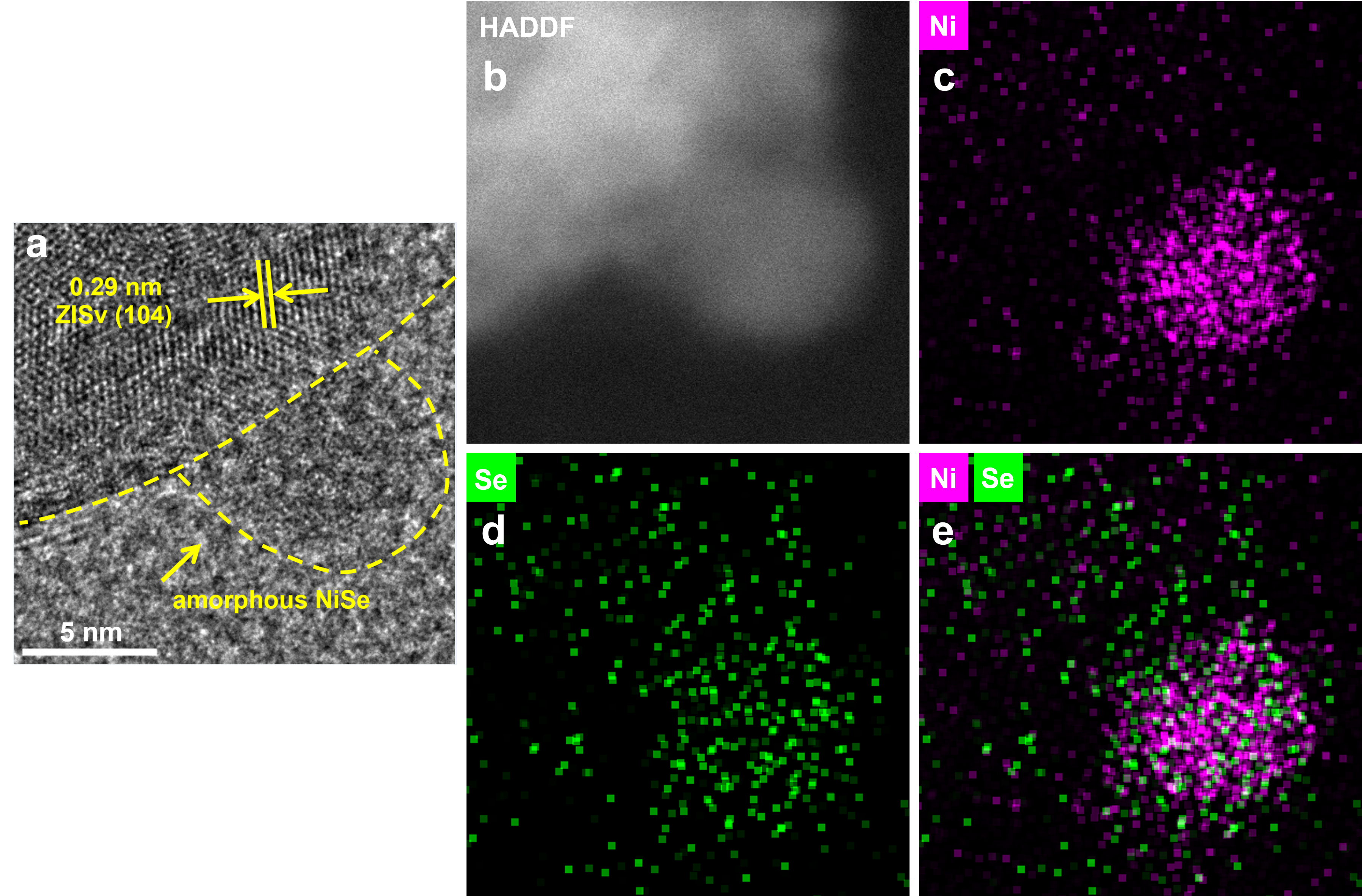


**Figure. S2.** (a, b) HRTEM image (a) and HAADF-STEM image (b) of the ZISv-NiSe sample. (c-e) EDX mapping images showing the distribution of Ni (c) and Se (d) elements, and the overlap of these two elements (e).


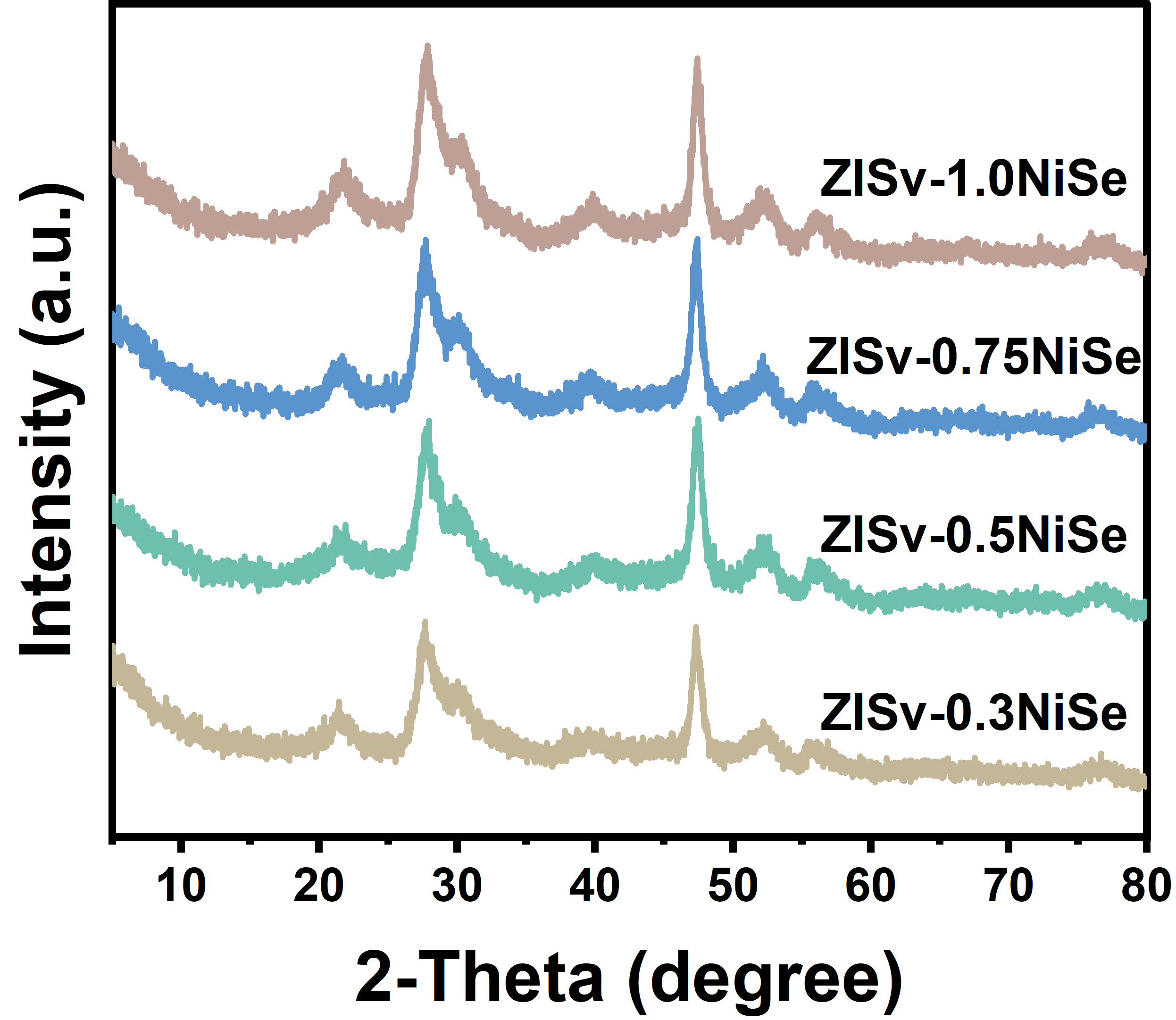


**Figure S3.** XRD patterns of different ZISv-xNiSe samples. where x represents the loading percent of Ni.


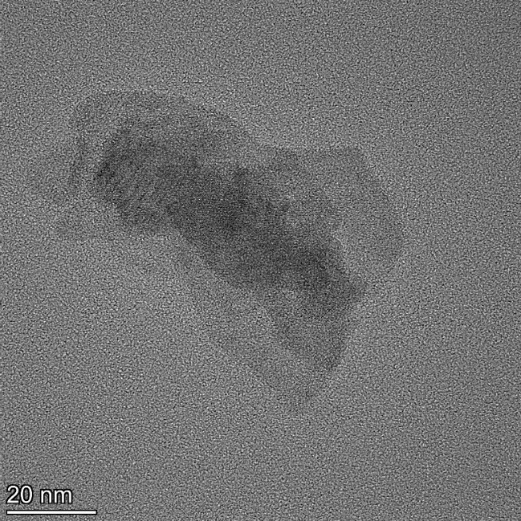


**Figure S4.** TEM image of the ZIS sample.


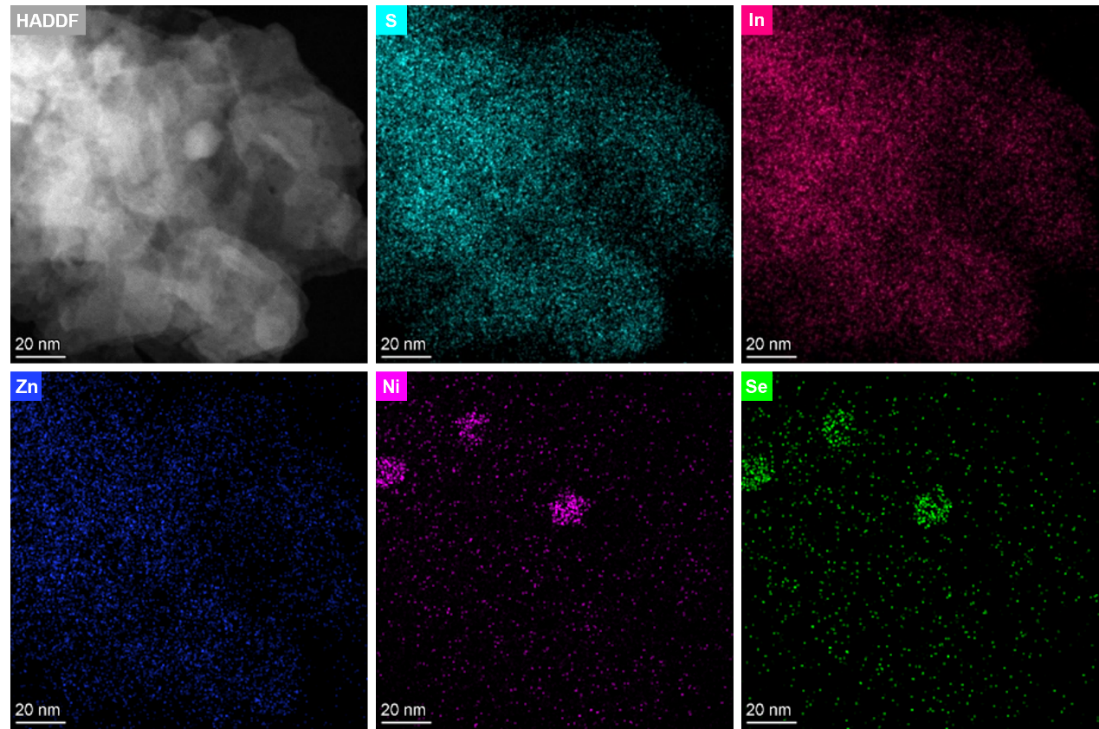


**Figure S5.** HAADF-STEM and the corresponding elemental mapping images of the ZIS-NiSe sample.


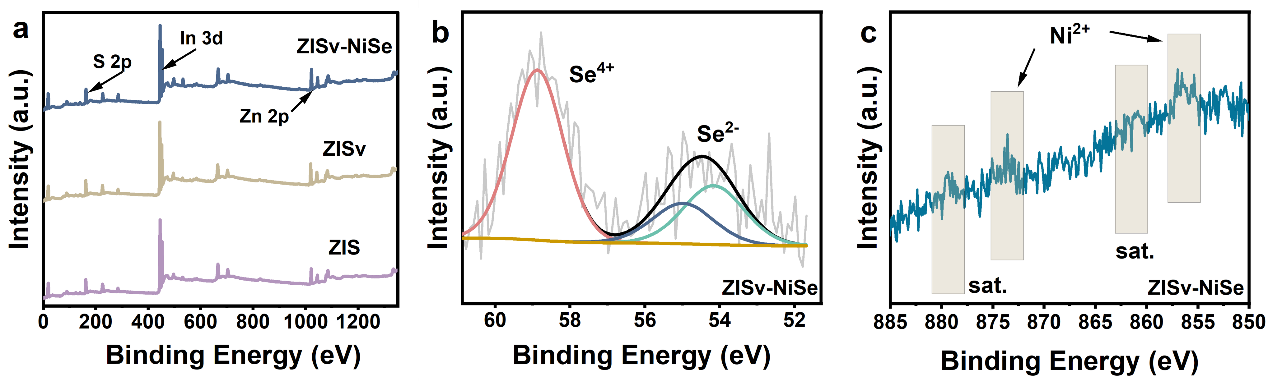


**Figure S6.** XPS spectra of (a) survey, (b) Se 3d and (c) Ni 2p for the ZISv-NiSe sample.


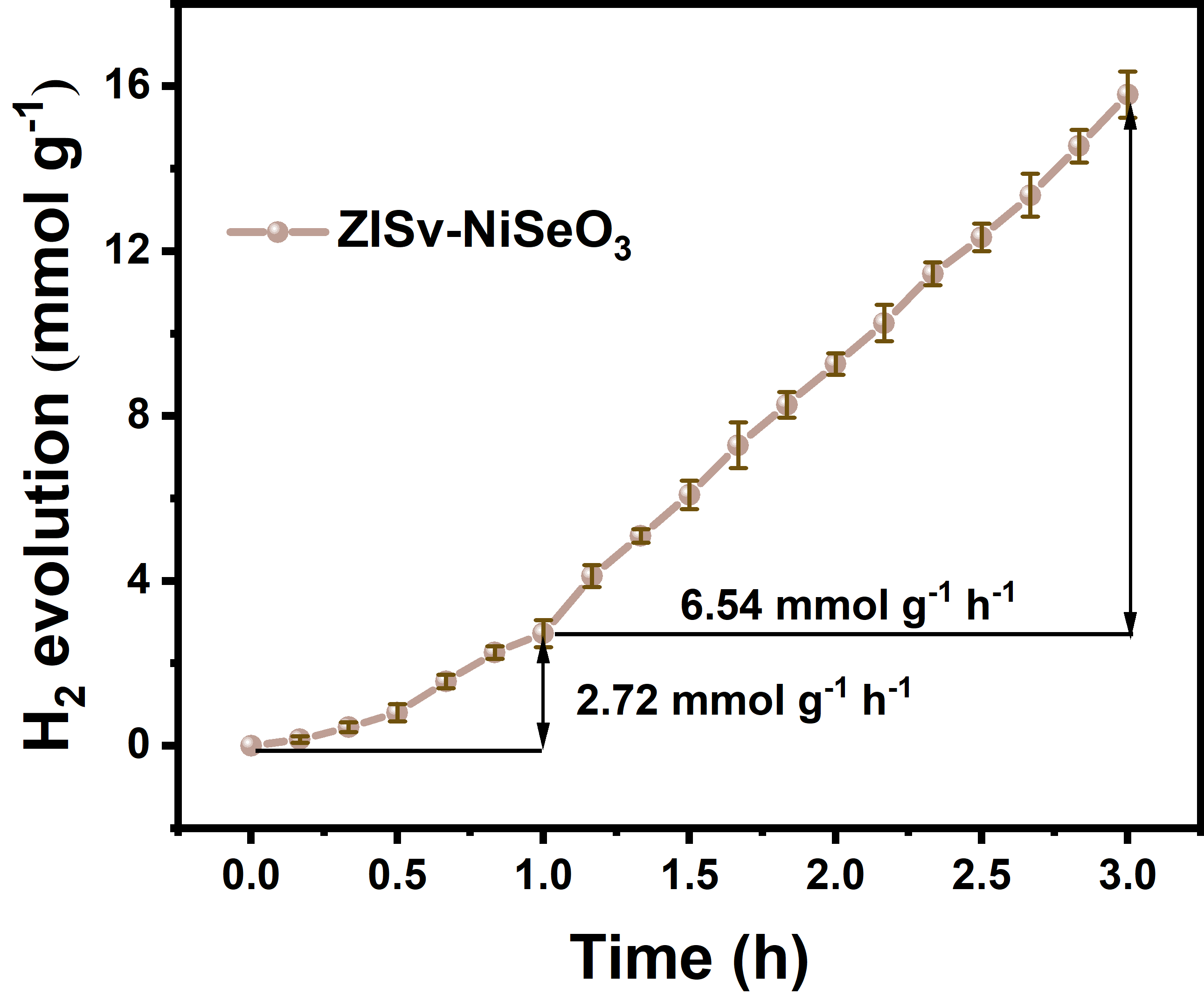


**Figure S7.** Plots illustrating the H_2_ evolution reaction at different time points for the ZISv-NiSeO_3_ sample.


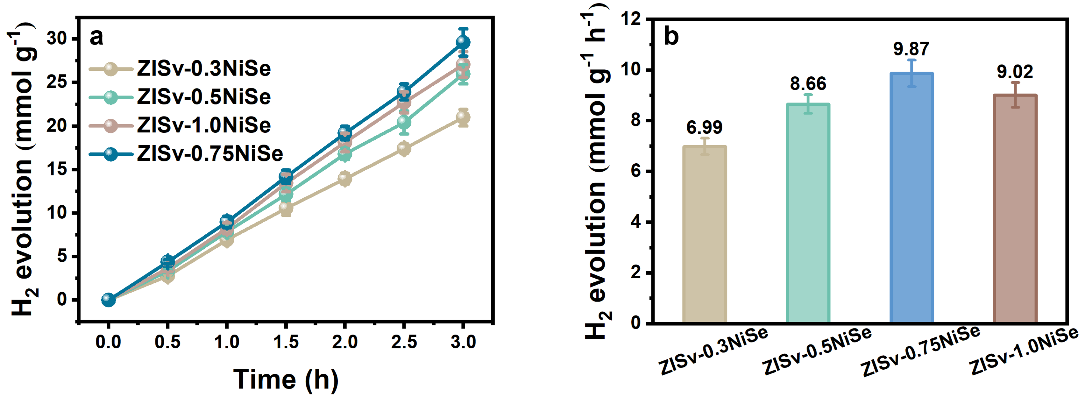


**Figure S8.** (a) Plots illustrating the H_2_ evolution reaction at different time points for different samples. (b) Histogram comparing the calculated H_2_ evolution rates for different samples.


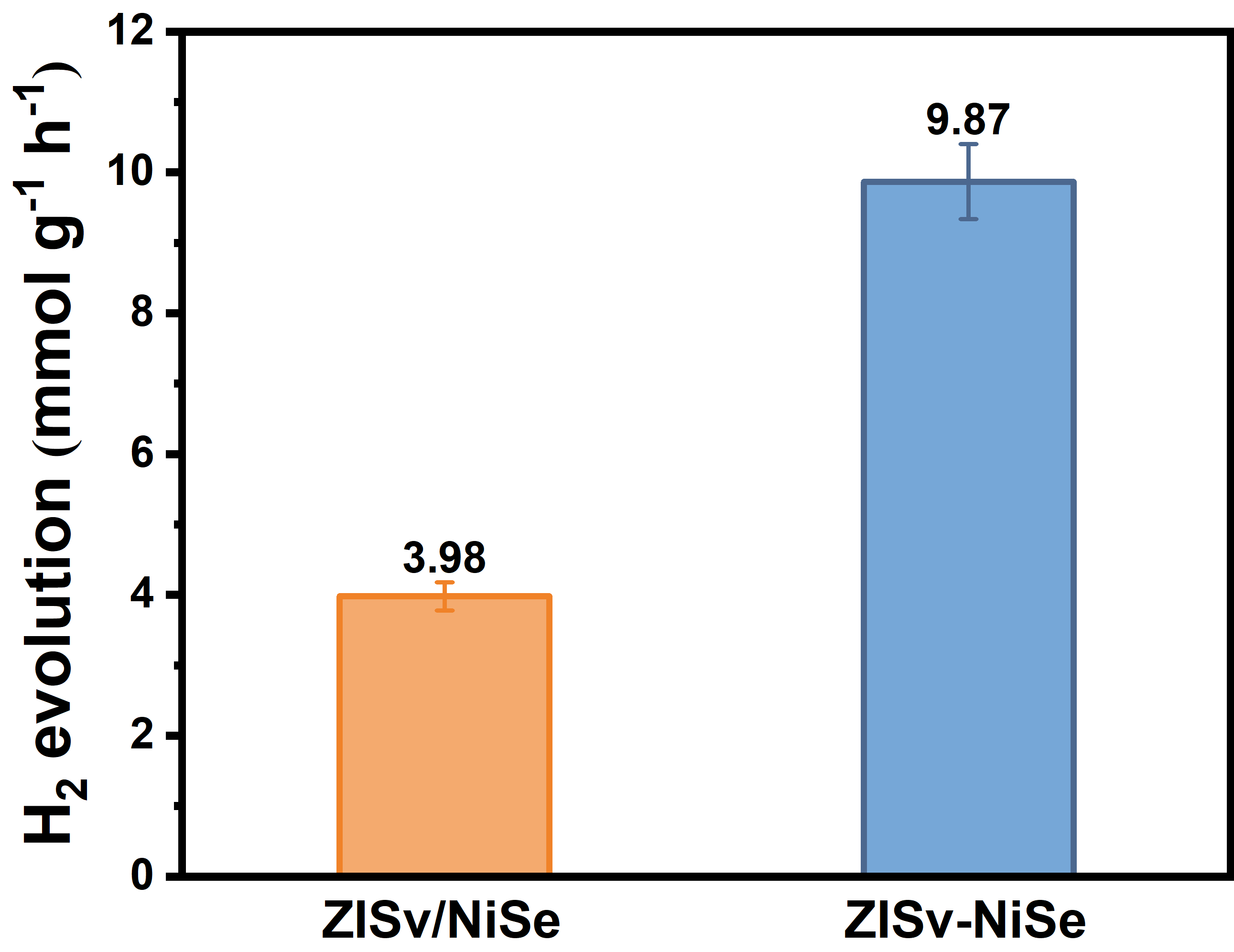


**Figure S9.** Histogram comparing the H_2_ evolution rates of the ZISv-NiSe sample and the physical mixture of ZISv and NiSe (termed as ZISv/NiSe).


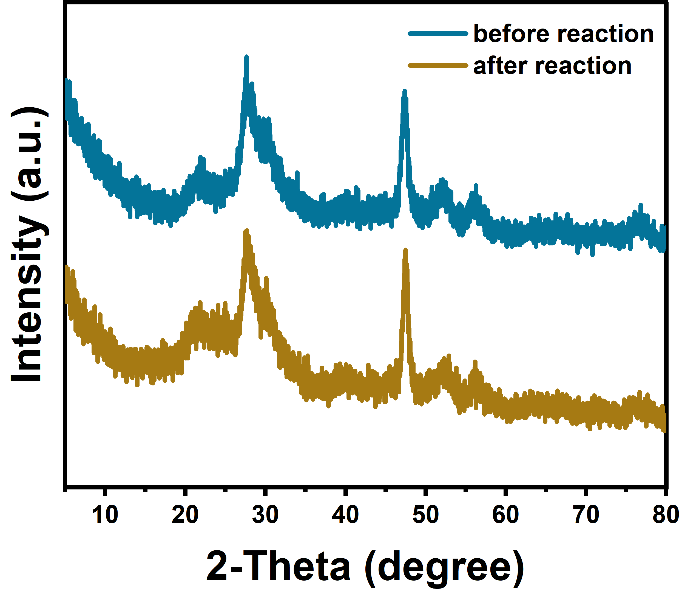


**Figure S10.** XRD patterns of the ZISv-NiSe sample before and after reaction.


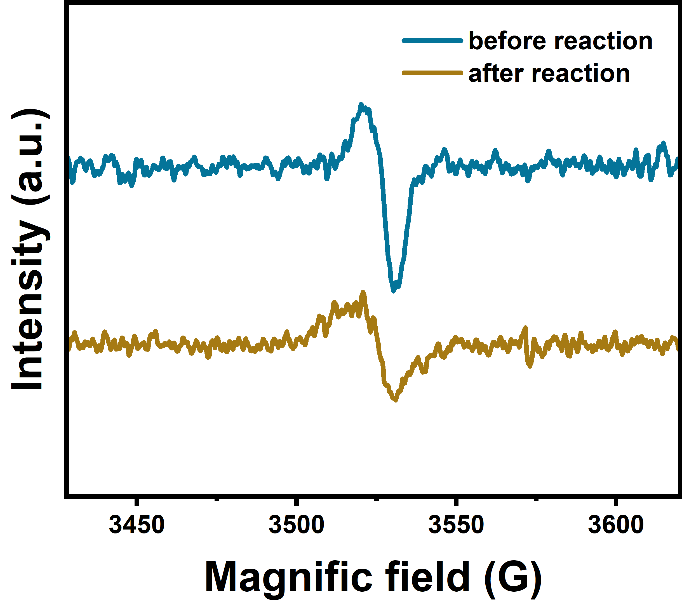


**Figure S11.** EPR spectra of the ZISv-NiSe sample before and after reaction.


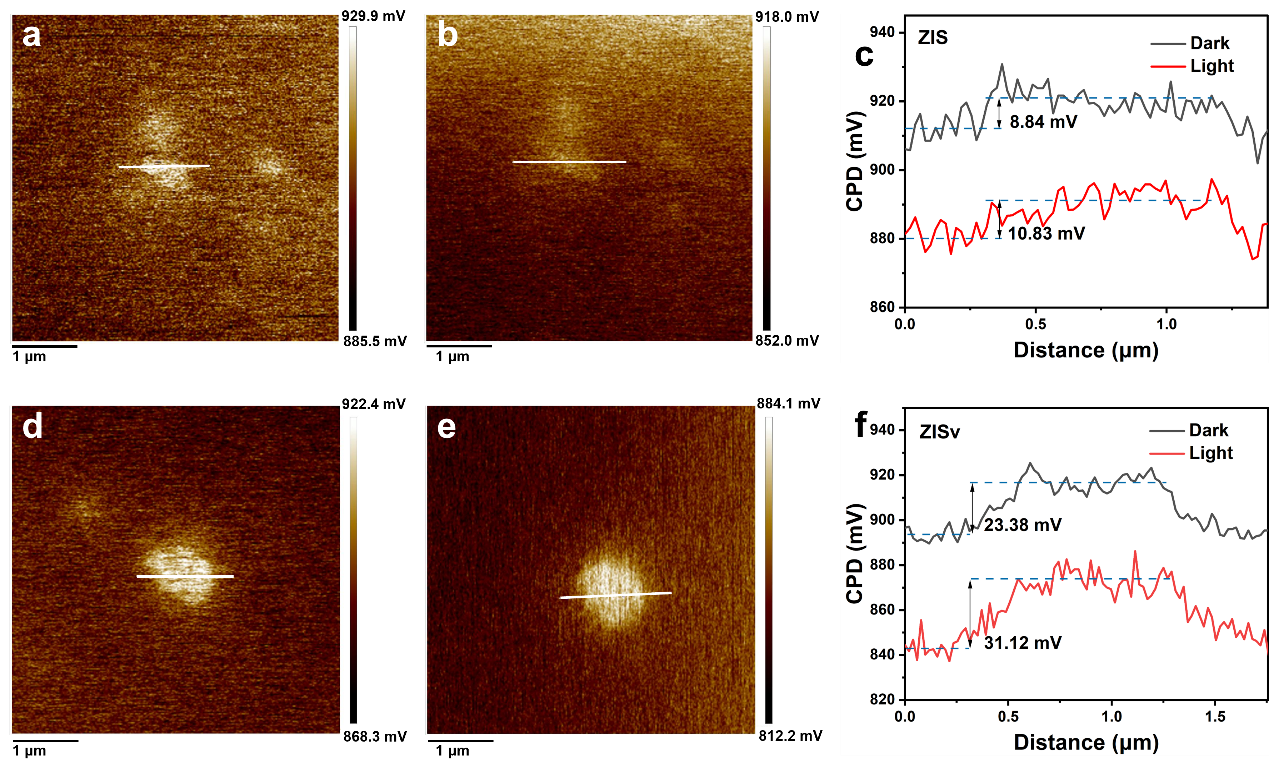


**Figure S12.** (a, b) Surface potential distribution of the ZIS sample under the dark (a) and light (b) conditions. (c) Surface potential across the line shown in (a) and (b). (d, e) Surface potential distribution of the ZISv sample under the dark (d) and light (e) conditions. (f) Surface potential across the line shown in (d) and (e).


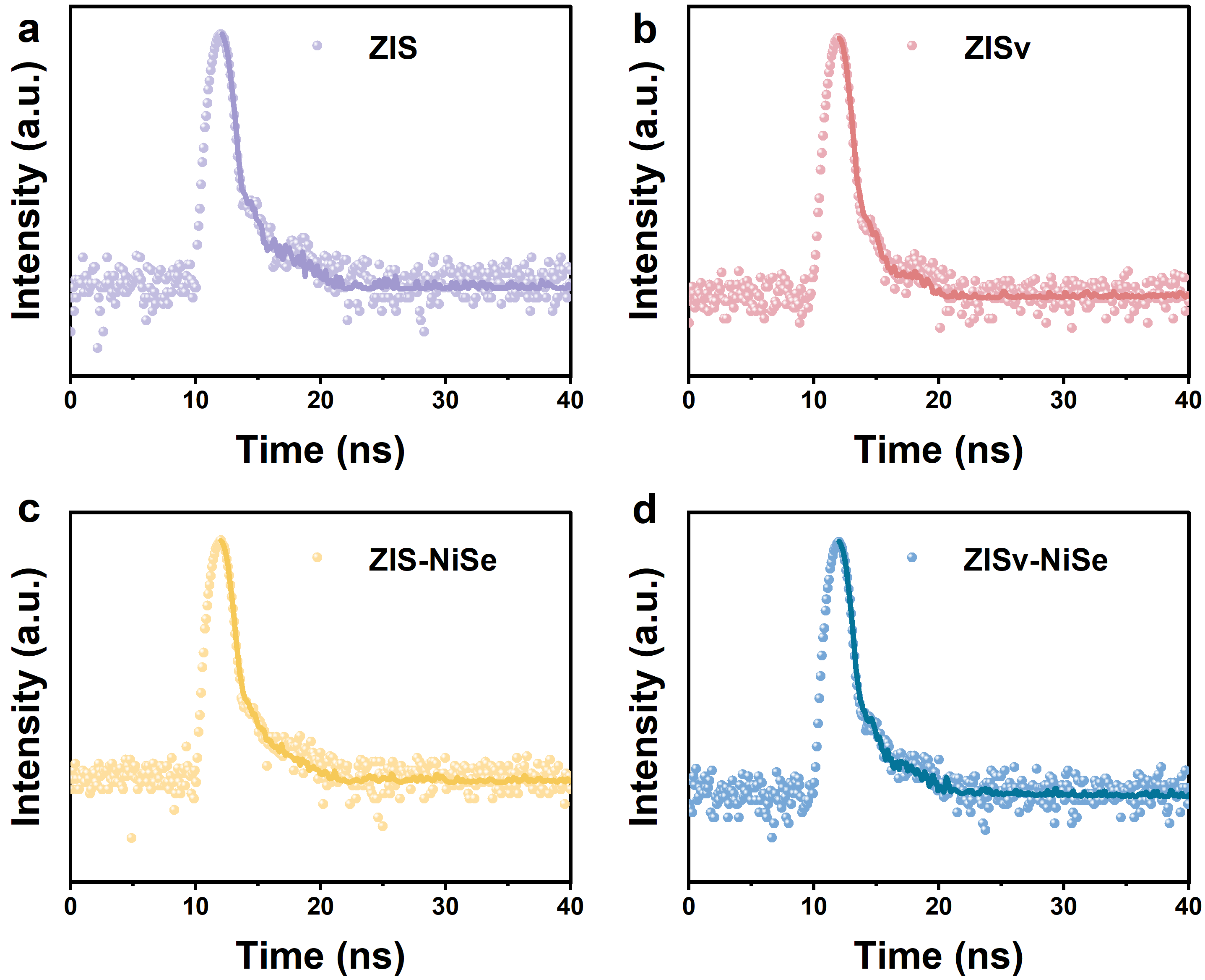


**Figure S13.** Time-resolved photoluminescence spectra for the ZIS, ZISv, ZIS-NiSe, and ZISv-NiSe samples.


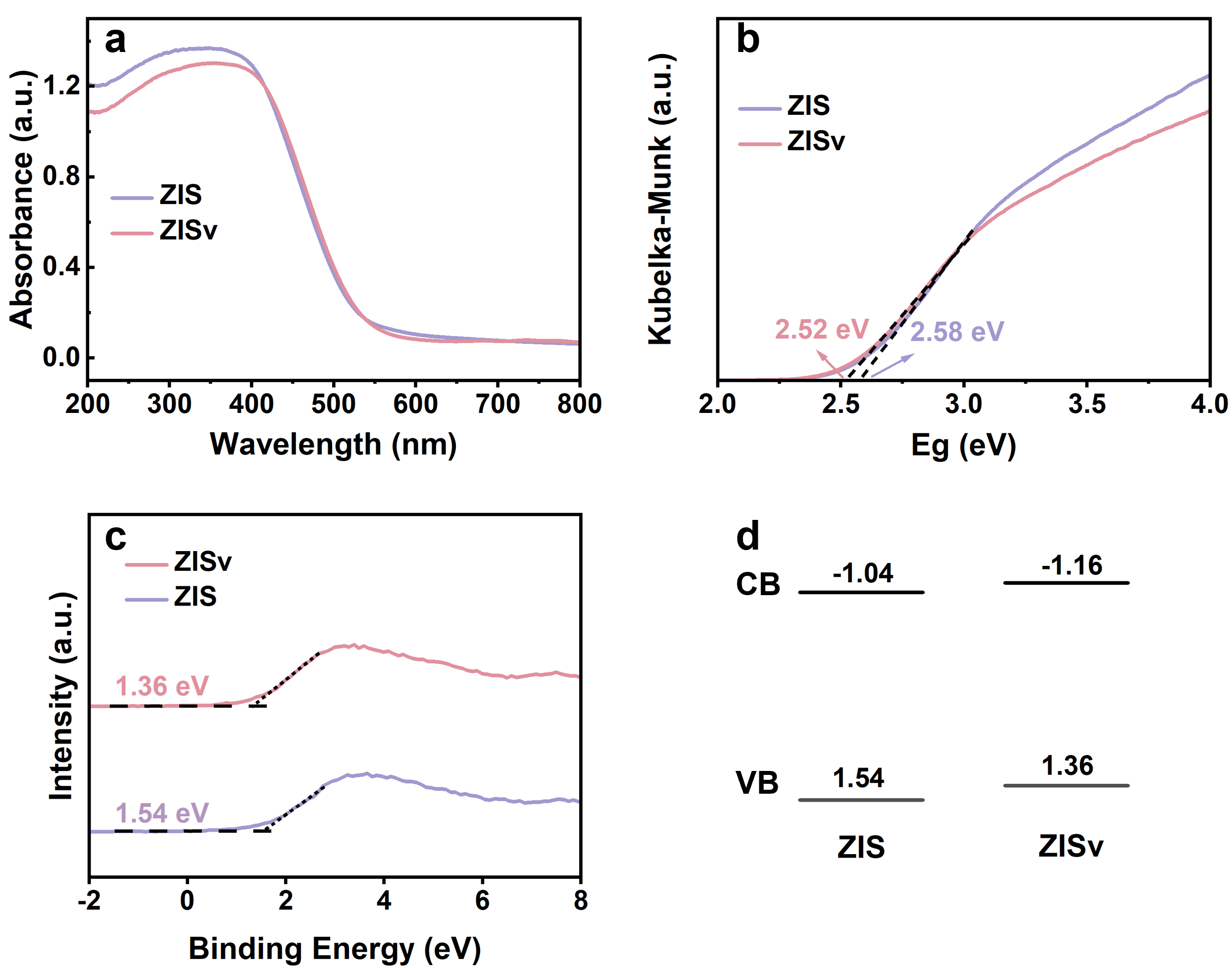


**Figure S14.** (a) UV-vis diffuse reflection spectra of the ZIS and ZISv samples. (b) Kubelka-Munk function vs. the energy of incident light plots. (c) Valence-band XPS spectra the ZIS and ZISv samples. (d) Schematic illustrating the band structures of the ZIS and ZISv samples


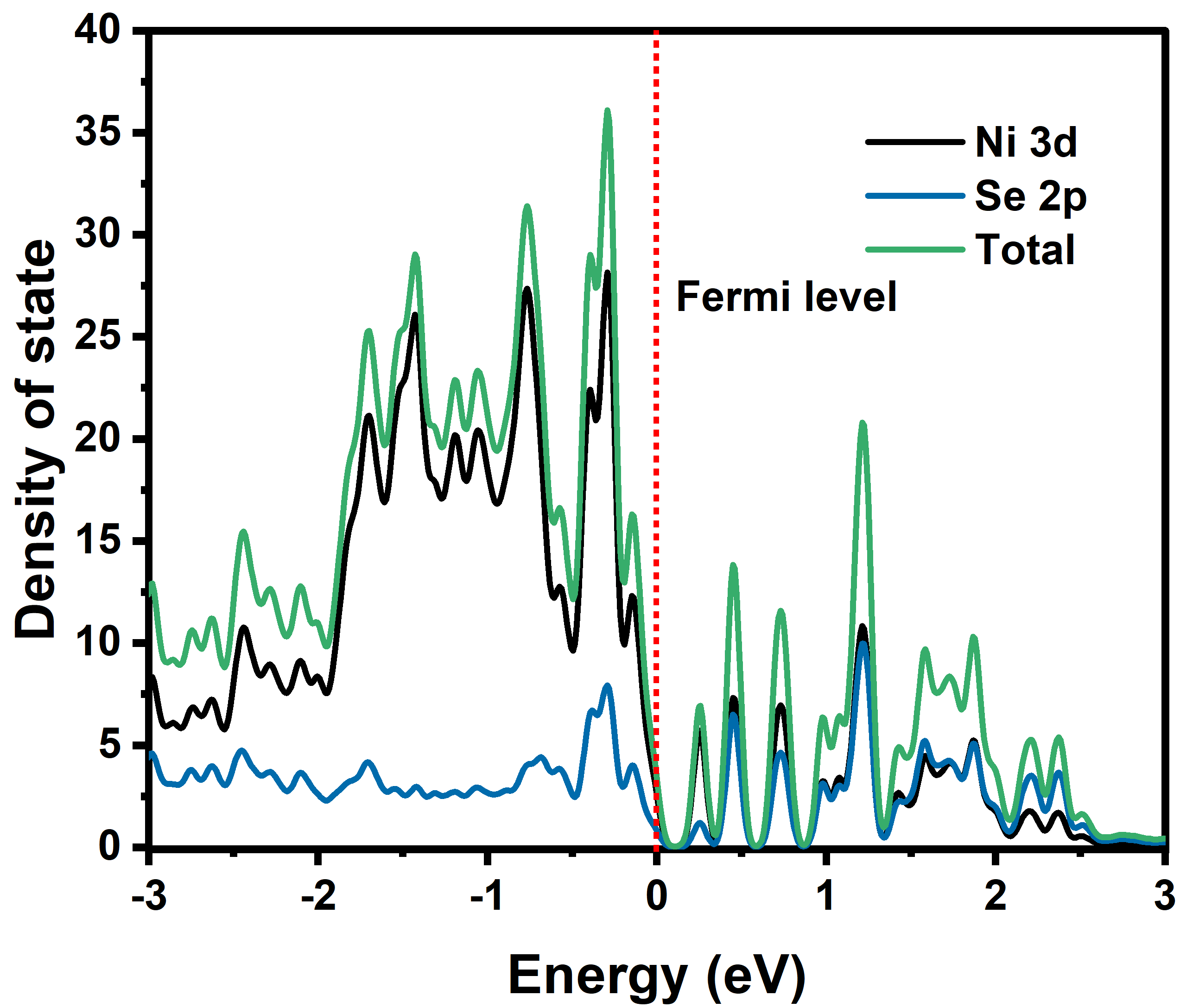


**Figure S15.** Calculated density of states for NiSe.


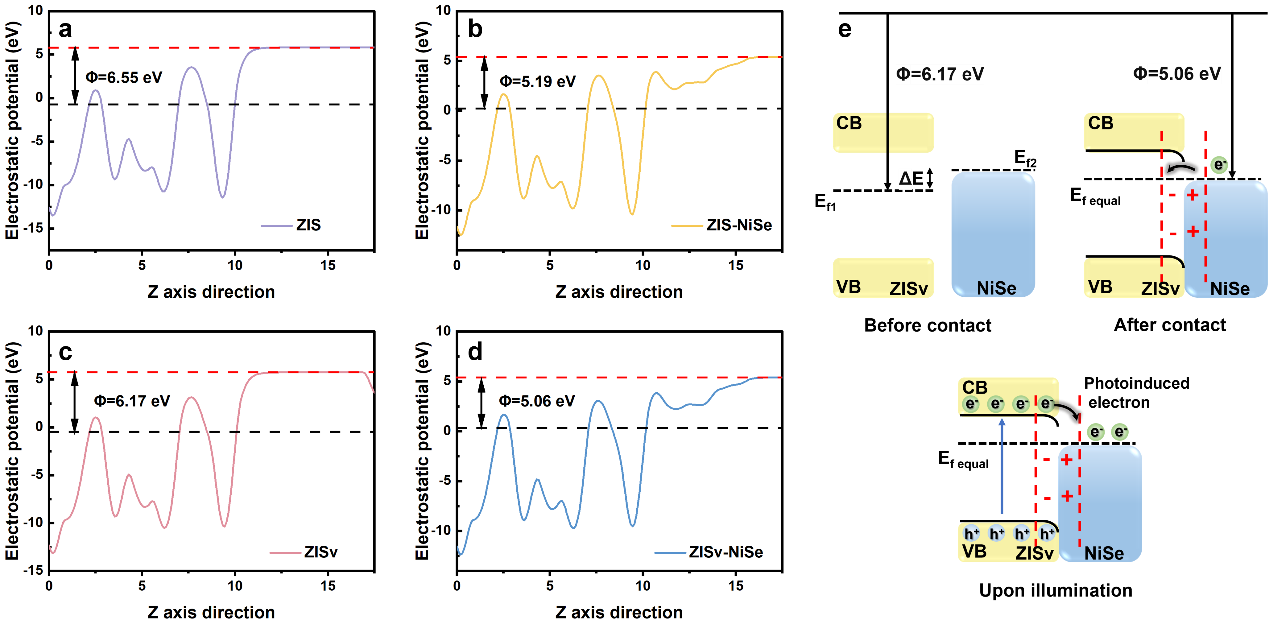


**Figure S16.** (a-d) Calculated average potential profile along the *z*-axis for the (a) ZIS, (b) ZIS-NiSe, (c) ZISv, and (d) ZISv-NiSe samples. (e) Illustration of the band structures of ZISv and NiSe before contact, after contact, and upon illumination.


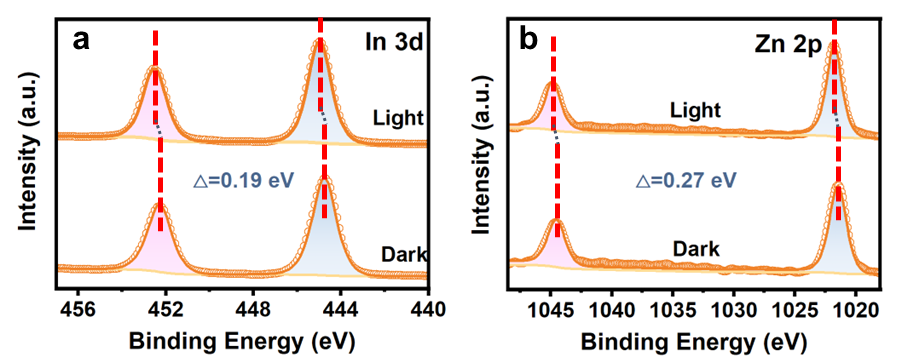


**Figure S17.** In situ XPS spectra of (a) In 3d and (b) Zn 2p for the ZISv-NiSe sample in the dark and under UV-light irradiation.


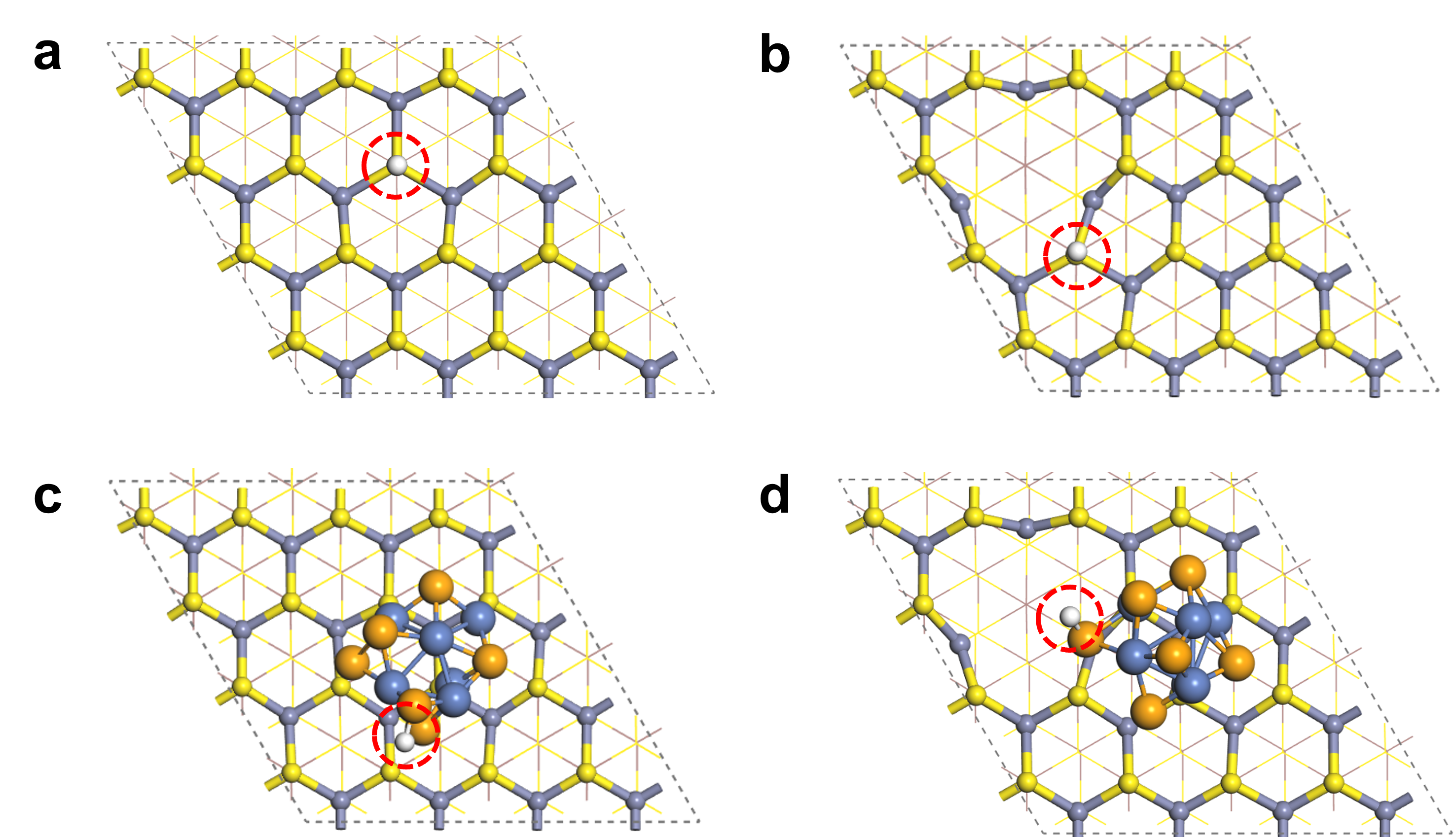


**Figure S18**. The models of H* adsorption sites on the surface of (a) ZIS, (b) ZISv, (c) ZIS-NiSe and (d) ZISv-NiSe.


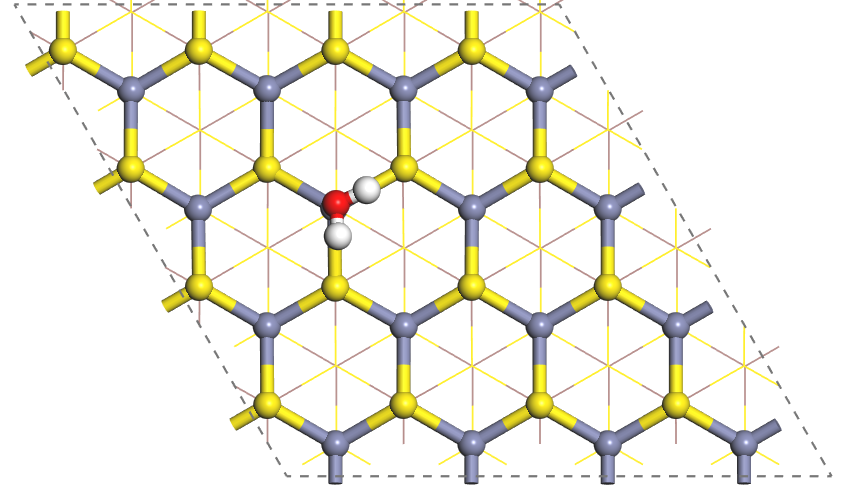


$\Delta G_{H_{2}O*}=0.00$ eV

**Figure S19.** Optimized configuration for H_2_O adsorption on ZIS.


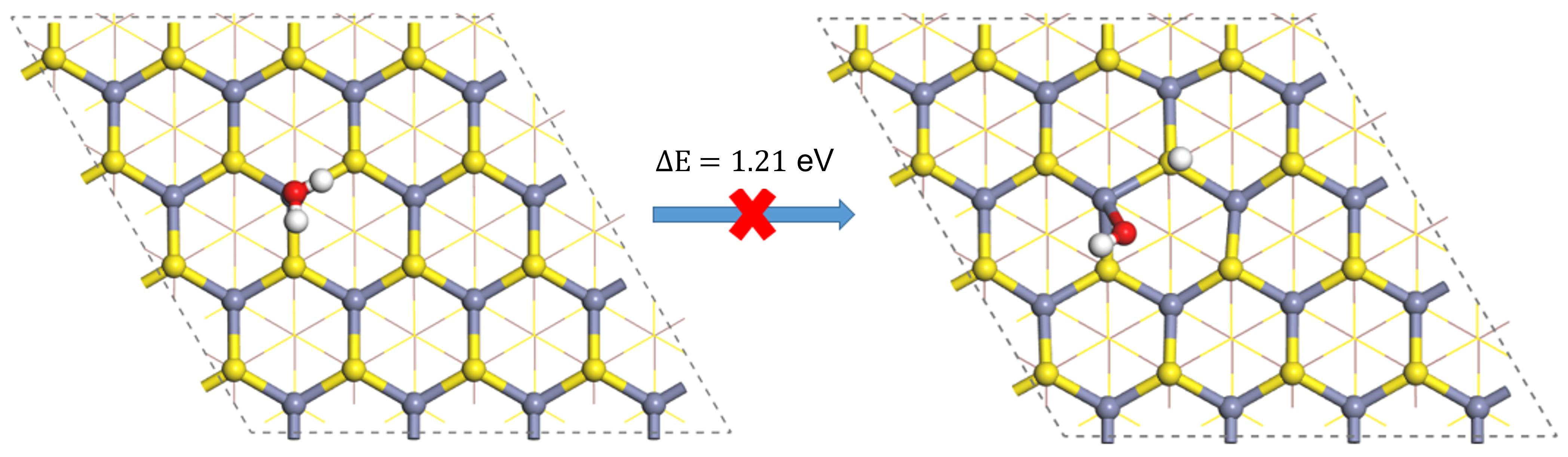


**Figure S20.** DFT optimized initial state and final state structures for H_2_O dissociation on ZIS.


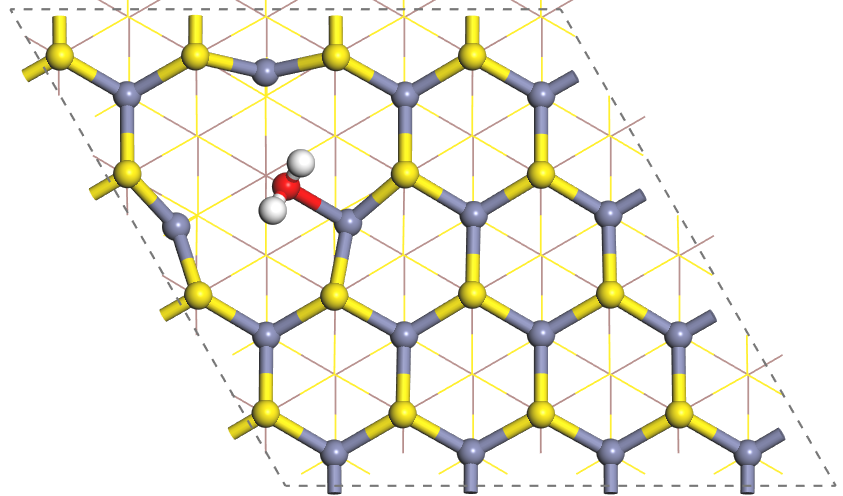


$\Delta G_{H_{2}O*}=-0.34$ eV

**Figure S21.** Optimized configuration for H_2_O adsorption on ZISv.


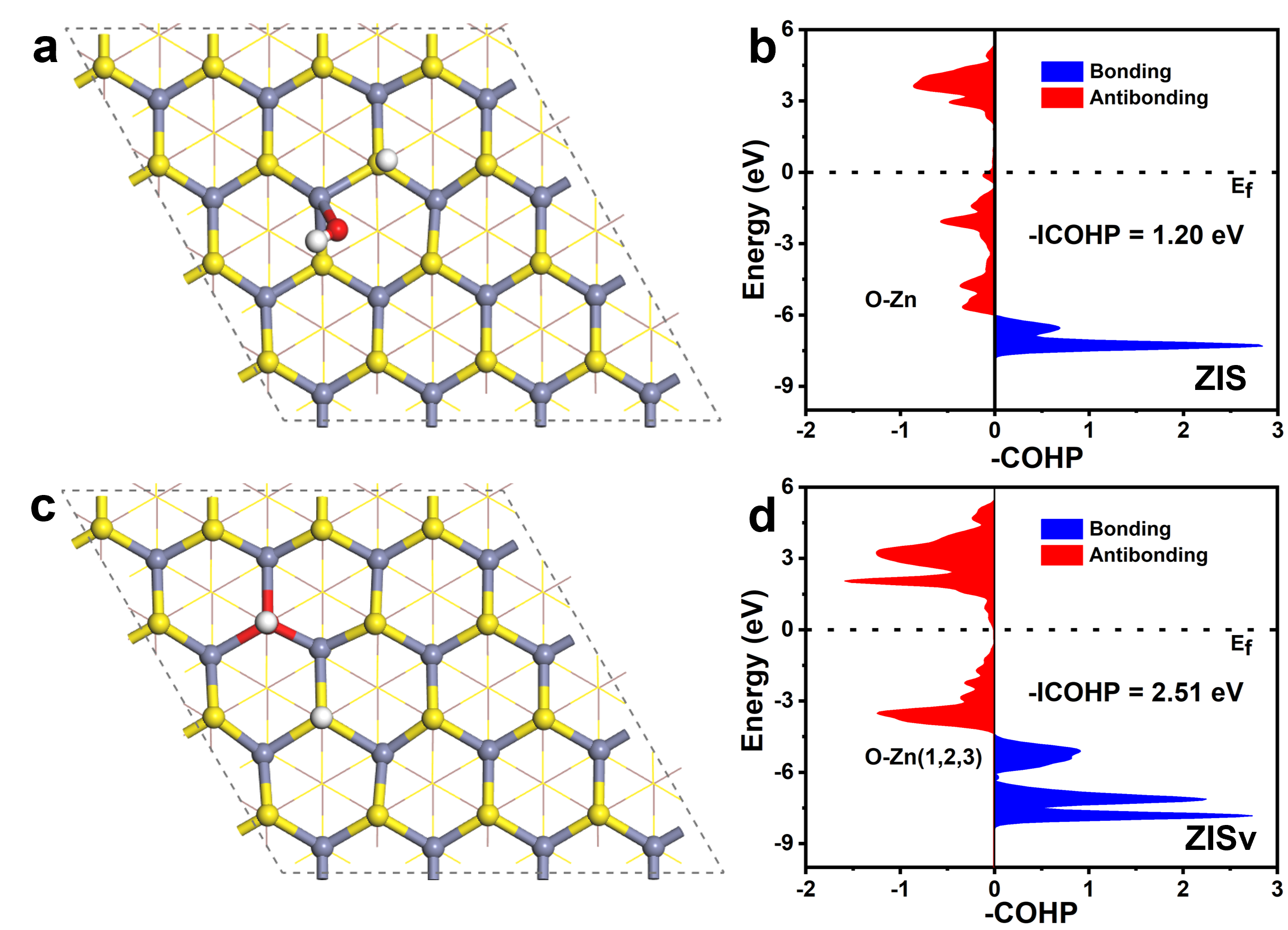


**Figure S22.** DFT optimized final state structures for H_2_O dissociation on (a) ZIS and (c) ZISv. (b) and (d) COHP analysis of the O-Zn bond for (b) ZIS and (d) ZISv.


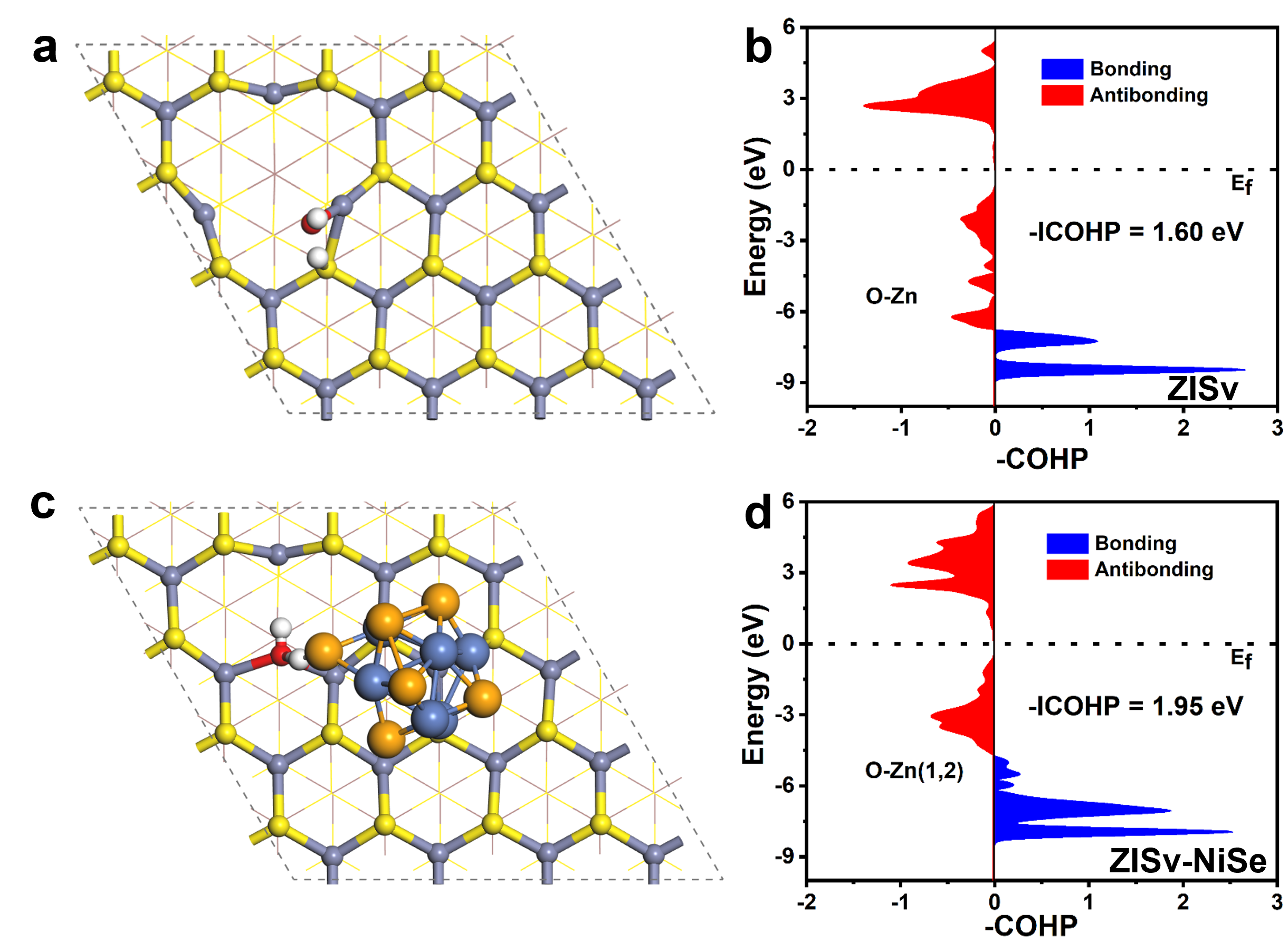


**Figure S23.** DFT optimized transition state structures for H_2_O dissociation on (a) ZISv and (c) ZISv-NiSe. (b) and (d) COHP analysis of the O-Zn bond for (b) ZISv and (d) ZISv-NiSe.


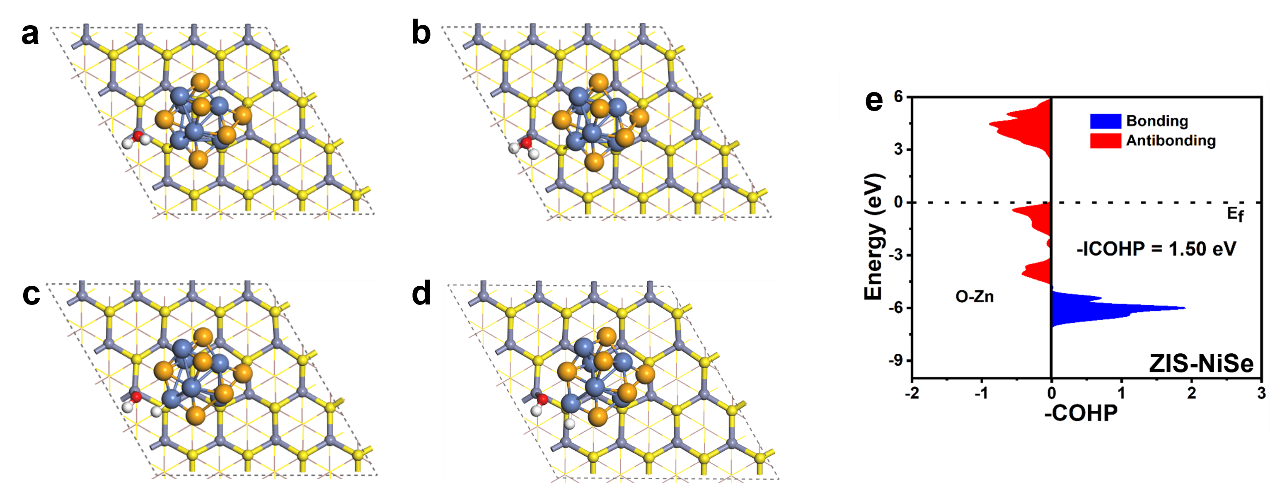


**Figure S24.** (a-d) Optimized structures for H_2_O dissociation taking place on the ZIS (001) surface in the presence of NiSe. (e) COHP analysis of the O-Zn bond for ZIS-NiSe of DFT optimized final state structures for H_2_O dissociation (d).


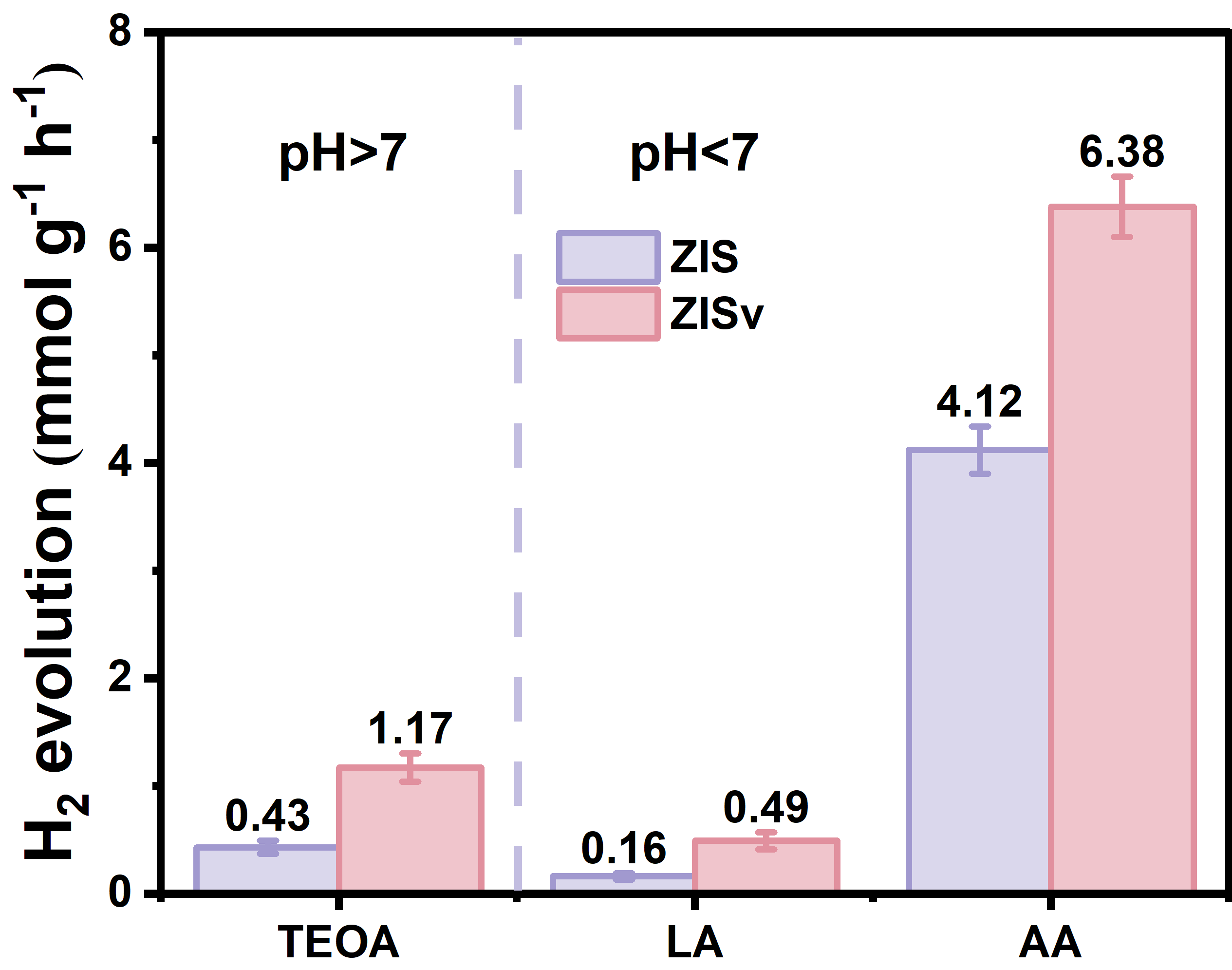


**Figure S25.** Histogram comparing the activity of the ZIS and ZISv samples under different pH conditions.


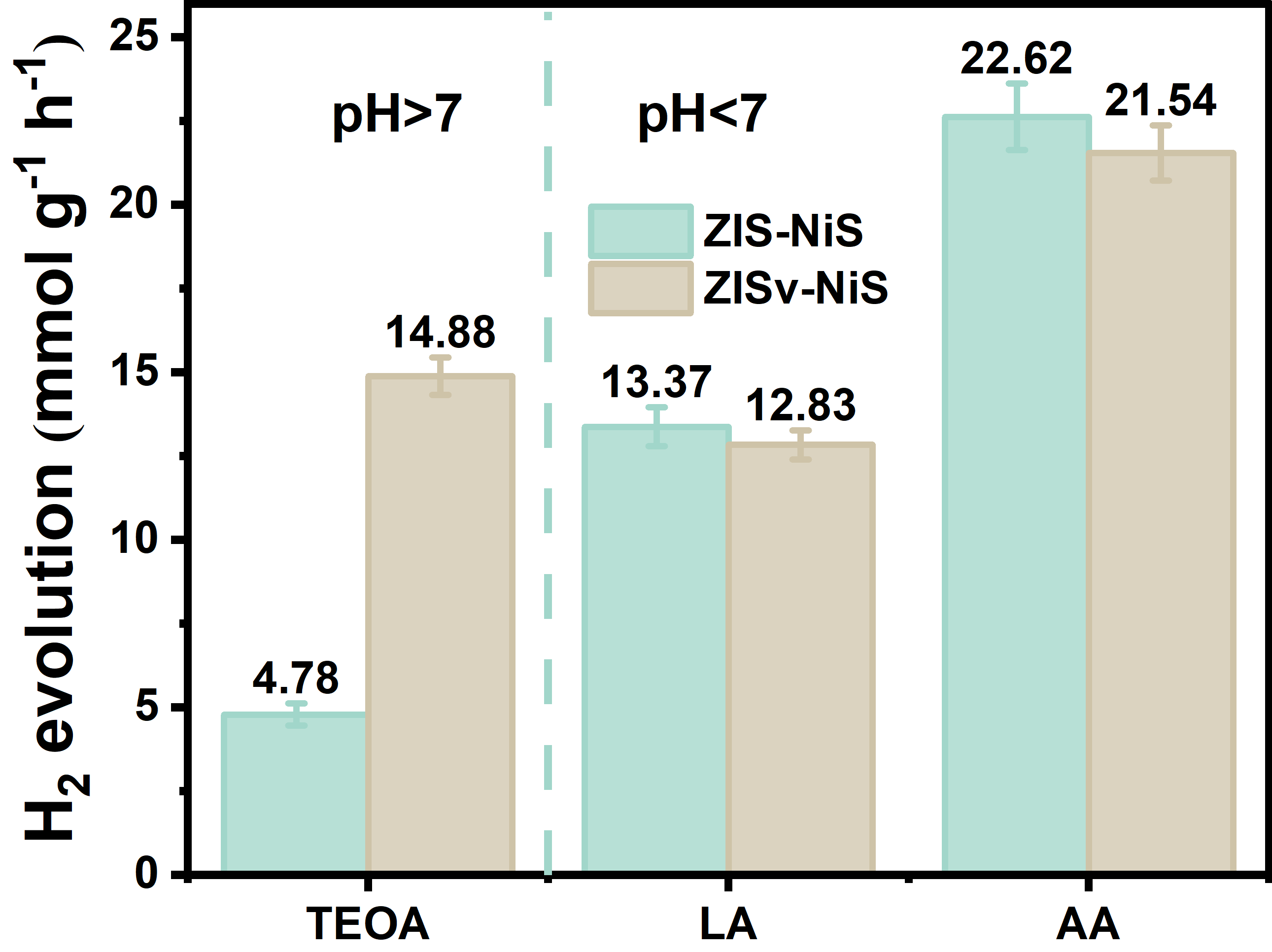


**Figure S26.** Histogram comparing the activity of the ZIS-NiS and ZISv-NiS samples at different pH conditions using different sacrificial donors.

**Table S1.** The calculated AQY of ZISv-NiSe photocatalyst at different wavelengths.

| **Wavelength**  **(nm)** | **The mean H_2_ production in 1 hour (μmol)** | **Light power**  **(W)** | **AQY** |
| --- | --- | --- | --- |
| 365 | 142.6 | 0.163 | 16.87% |
| 420 | 96.0 | 0.158 | 9.15% |
| 475 | 22.5 | 0.168 | 1.88% |
| 520 | 1.1 | 0.179 | 0.08% |

**Table S2.** Comparison of the activity of ZISv-NiSe with other similar state-of-the-art photocatalysts in photocatalytic hydrogen evolution.

| **Photocatalysts** | **Light source** | **Sacrificial**  **agents** | **H_2_ evolution rate (mmol h^-1^ g^-1^)** | **AQY** | **Ref.** |
| --- | --- | --- | --- | --- | --- |
| Ni_0.7_Co_0.3_P/ZIS | 300 W Xe lamp  (λ > 420 nm) | TEOA | 3.84 | 3.39% (420 nm) | ^[5]^ |
| MoS_2_/O-ZnIn_2_S_4_ | 300 W Xe lamp  (λ > 420 nm) | Na_2_S/Na_2_SO_3_ | 4.00 | 2.53% (420nm) | ^[6]^ |
| COF/ZIS-20% | 300 W Xe lamp  (λ > 420 nm) | Na_2_S/Na_2_SO_3_ | 0.85 | 2.08% (420nm) | ^[7]^ |
| ZnIn_2_S_4_@Mxene (3%Pt) | 300 W Xe lamp  (λ > 420 nm) | TEOA | 3.48 | not provided | ^[8]^ |
| WS_2_/ZnIn_2_S_4_ | 300 W Xe lamp  (λ > 420 nm) | Lactic acid | 2.55 | 3.2% (420nm) | ^[9]^ |
| NCSC/ZnIn_2_S_4_ | 300 W Xe lamp  (λ > 420 nm) | TEOA | 5.10 | 4.35% (420nm) | ^[10]^ |
| SB-ZIS-2(3%Pt) | 300 W Xe lamp | TEOA | 1.69 | 3.8% (420nm) | ^[11]^ |
| COP-ZIS  COP-ZIS (Pt) | 300 W Xe lamp | Na_2_S/Na_2_SO_3_ | 0.95  5.04 | not provided | ^[12]^ |
| 0.15 ZIS/TiO_2-x_ | 300 W Xe lamp | MeOH | 0.58 | 1.42% (380 nm) | ^[13]^ |
| TiM@ZIS/CdS | 300 W Xe lamp  (AM 1.5) | MeOH | 2.37 | not provided | ^[14]^ |
| BC/ZIS | 300 W Xe lamp  (AM 1.5) | Lactic acid | 4.47 | 0.45% (420nm) | ^[15]^ |
| ZISv-NiSe | 300 W Xe lamp  (AM 1.5) | TEOA | 9.65 | 9.15% (420 nm) | This work |

**Table S3.** Fitted parameters for the time-resolved photoluminescence spectra of the ZIS, ZISv, ZIS-NiSe, and ZISv-NiSe samples.

| **Samples** | **τ_1_(ns)** | **Rel_1_%** | **τ_2_(ns)** | **Rel_2_%** | **τ(ns)** |
| --- | --- | --- | --- | --- | --- |
| ZIS | 0.035 | 99.89 | 24.21 | 0.11 | 0.062 |
| ZISv | 0.052 | 99.93 | 37.83 | 0.07 | 0.078 |
| ZIS-NiSe | 0.016 | 99.69 | 22.02 | 0.31 | 0.084 |
| ZISv-NiSe | 0.01 | 99.67 | 30.08 | 0.33 | 0.109 |

References:

[1] B. Ravel, M. Newville, *J. Synchrotron Radiat.* **2005**, *12*, 537-541.

[2] G. Kresse, J. Furthmüller, *Phys rev B* **1996**, *54*, 11169.

[3] J. P. Perdew, A. Ruzsinszky, G. I. Csonka, O. A. Vydrov, G. E. Scuseria, L. A. Constantin, X. Zhou, K. Burke, *Phys. Rev. Lett.* **2008**, *100*, 136406.

[4] a) S. Grimme, J. Antony, S. Ehrlich, H. Krieg, *J. Chem. Phys.* **2010**, *132*, 154104; b) S. Grimme, S. Ehrlich, L. Goerigk, *J. Comput. Chem.* **2011**, *32*, 1456-1465.

[5] H. Song, S. Liu, Z. Sun, Y. Han, J. Xu, Y. Xu, J. Wu, H. Meng, X. Xu, T. Sun, X. Zhang, *Sep. Purif. Technol.* **2021**, *275*, 119153.

[6] Y. Peng, X. Guo, S. Xu, Y. n. Guo, D. Zhang, M. Wang, G. Wei, X. Yang, Z. Li, Y. Zhang, F. Tian, *J. Energy Chem.* **2022**, *75*, 276-284.

[7] P. Dong, T. Cheng, J.-l. Zhang, J. Jiang, L. Zhang, X. Xi, J. Zhang, *ACS Appl. Energy Mater.* **2023**, *6*, 1103-1115.

[8] G. Zuo, Y. Wang, W. L. Teo, A. Xie, Y. Guo, Y. Dai, W. Zhou, D. Jana, Q. Xian, W. Dong, Y. Zhao, *Angew. Chem. Int. Ed.* **2020**, *59*, 11287-11292.

[9] M. Xiong, B. Chai, J. Yan, G. Fan, G. Song, *Appl. Surf. Sci.* **2020**, *514*, 145965.

[10] Y. Chao, P. Zhou, J. Lai, W. Zhang, H. Yang, S. Lu, H. Chen, K. Yin, M. Li, L. Tao, C. Shang, M. Tong, S. Guo, *Adv. Funct. Mater.* **2021**, *31*, 2100923.

[11] Y. Xiao, H. Wang, Y. Jiang, W. Zhang, J. Zhang, X. Wu, Z. Liu, W. Deng, *J. Colloid Interface Sci.* **2022**, *623*, 109-123.

[12] C. Cui, X. Zhao, X. Su, N. Xi, X. Wang, X. Yu, X. L. Zhang, H. Liu, Y. Sang, *Adv. Funct. Mater.* **2022**, *32*, 2208962.

[13] D. Zhang, M. Wang, G. Wei, R. Li, N. Wang, X. Yang, Z. Li, Y. Zhang, Y. Peng, *Appl. Surf. Sci.* **2023**, *622*, 156839.

[14] S. Zhang, M. Du, Z. Xing, Z. Li, K. Pan, W. Zhou, *Appl Catal B-environ* **2020**, *262*, 118202.

[15] P. Bhavani, D. Praveen Kumar, M. Hussain, T. M. Aminabhavi, Y.-K. Park, *Chem. Eng. J.* **2022**, *434*, 134743.
